# Supplementary material for: Association between early methadone dose titration and treatment discontinuation and opioid toxicity: A retrospective cohort study
Source: PLoS Med. 2026 Apr 9;23(4):e1004748. doi: 10.1371/journal.pmed.1004748 (PMC13065010; doi:10.1371/journal.pmed.1004748)
Supplement: S2 Table — (DOCX) [file pmed.1004748.s002.docx]

**S2 Table.** Descriptions of all linked administrative databases used in the study

| **Database** | **Description** |
| --- | --- |
| Narcotics Monitoring System (NMS) | Captures all prescriptions for controlled substances dispensed from community pharmacies in Ontario, regardless of insurance status. This database was used to identify opioid agonist therapy dispensations. |
| Registered Persons Database (RPDB) | Captures demographic and vital status characteristics for everyone eligible for the publicly funded Ontario Health Insurance Plan (OHIP). |
| Canadian Institute for Health Information (CIHI) Discharge Abstract Database (DAD) | Captures details on diagnoses and procedures for all inpatient hospital stays in Ontario. |
| CIHI National Ambulatory Care Reporting System (NACRS) | Captures details on diagnoses and procedures for all emergency department visits in Ontario. |
| CIHI Ontario Mental Health Reporting System (OMHRS) | Captures details on diagnoses and procedures for all inpatient stays in mental health hospitals in Ontario. |
| Ontario Health Insurance Plan (OHIP) Database | Captures outpatient care using billing information for all services covered by OHIP in Ontario. |
| Ontario Drug Benefit Claims (ODB) | Captures all prescriptions dispensed through the Ontario Drug Benefit program. |
| CIHI-Same Day Surgery (CIHI-SDS) | Patient-level demographic, diagnostic, procedural and treatment information on all day surgeries |
| Community Health Centre (CHC) | Records pertaining to outpatient primary care provider visits at a CHC. |
| ICES Physician Database (IPDB) | Captures information on physicians eligible to receive payment from the OHIP, such as demographics, training, and practice location. |
| Ontario Cancer Registry (OCR) | A provincial registry of all incident cancer diagnoses in Ontario. |
| Cancer Activity Level Reporting (ALR) | Captures details regarding inpatient and outpatient provision of radiation and systemic therapy services for cancer treatment. |
| New Drug Funding Program (NDFP) | Captures prescription for expensive drugs (e.g., outpatient cancer medications) reimbursed by the provincial drug program. |
| MOMBABY | MOMBABY is an ICES-created database. It includes 100% of Ontario inpatient birth admission records from the Discharge Abstract Database for mothers and their newborns. |
| HIV Validated Database | This database uses a validated definition to identify diagnosis dates for all people with HIV in Ontario (96.2% sensitivity, 99.6% specificity).^1^ |
| Ontario Asthma Dataset | This database uses a validated definition to identify diagnosis dates for all people with asthma in Ontario (83.8% sensitivity, 76.5% specificity).^2^ |
| Chronic Obstructive Pulmonary Disease (COPD) | Validated Database This database uses a validated definition to identify diagnosis dates for all people with COPD in Ontario (85.0% sensitivity, 78.4% specificity).^3^ |
| Drug-Alcohol Related Death Database | Contains details from investigations for all deaths occurring in Ontario where an investigating coroner has determined opioids to be direct contributors to death. |
| Primary Care Population (PCPOP) | Contains information pertaining to primary care rostering, such as which physician/group and family health team the patient is enrolled or virtually enrolled to. |

**REFERENCES:**

1. Antoniou T, Zagorski B, Loutfy MR, Strike C, Glazier RH. Validation of case-finding algorithms derived from administrative data for identifying adults living with human immunodeficiency virus infection. PLoS One. 2011;6(6):e21748. doi:10.1371/journal.pone.0021748

2. Gershon AS, Wang C, Guan J, Vasilevska-Ristovska J, Cicutto L, To T. Identifying patients with physician-diagnosed asthma in health administrative databases. Can Respir J. 2009;16(6):183-8. doi:10.1155/2009/963098

3. Gershon AS, Wang C, Guan J, Vasilevska-Ristovska J, Cicutto L, To T. Identifying individuals with physcian diagnosed COPD in health administrative databases. COPD. Oct 2009;6(5):388-94. doi:10.1080/15412550903140865
