# Supplementary material for: Association between early methadone dose titration and treatment discontinuation and opioid toxicity: A retrospective cohort study
Source: PLoS Med. 2026 Apr 9;23(4):e1004748. doi: 10.1371/journal.pmed.1004748 (PMC13065010; doi:10.1371/journal.pmed.1004748)
Supplement: S3 Table — (DOCX) [file pmed.1004748.s003.docx]

**S3 Table.** Censoring criteria applied to each outcome

|  | **Total** | **Death** | **Methadone discontinued** | **Opioid toxicity** | **Non-methadone toxicity** | **Methadone toxicity** |
| --- | --- | --- | --- | --- | --- | --- |
| **Intention to Treat Analysis** | | | |  | | |
| Opioid toxicity | N=13,560 | Censored (non-opioid death);  N=47 (0.35%) | - | Outcome; N=721 (5.32%) | - | - |
| Methadone discontinuation | N=13,560 | Censored (all-cause); N=25 (0.18%) | Outcome; N=8,385 (61.84%) | - | - | - |
| **While on Treatment** | | | |  | | |
| **Opioid toxicity** | N=13,560 | Censored (non-opioid death);  N=9 (0.07%) | Censored; N=8,213 (60.57%) | Outcome; N=225 (1.66%) | - | - |
| **Methadone toxicity** | N=13,560 | Censored (non-opioid death);  N=9 (0.07%) | Censored; N=8,213 (60.57%) | - | Censored; N=149 (1.10%) | Outcome; N=76 (0.56%) |
| **Non-methadone toxicity** | N=13,560 | Censored (non-opioid death);  N=9 (0.07%) | Censored; N=8,213 (60.57%) | - | Outcome; N=76 (0.56%) | Censored; N=149 (1.10%) |
