# Supplementary material for: Association between early methadone dose titration and treatment discontinuation and opioid toxicity: A retrospective cohort study
Source: PLoS Med. 2026 Apr 9;23(4):e1004748. doi: 10.1371/journal.pmed.1004748 (PMC13065010; doi:10.1371/journal.pmed.1004748)
Supplement: S4 Table — (DOCX) [file pmed.1004748.s004.docx]

**S4 Table.** Covariate definitions

| **Variable** | **Database** | **Definition** |
| --- | --- | --- |
| **Patient-related covariates** | | |
| Age at index | RPDB | Age at index using birth date in RPDB |
| Sex at index | RPDB | Sex as recorded in RPDB |
| Rurality of residence at index | RPDB | Rurality of residence using person’s postal code |
| Neighbourhood income quintile at index | RPDB | Neighbourhood income quintile using person’s postal code |
| Hospital flagged homelessness in the 365-days prior to index date | NACRS, DAD, OMHRS | Any ED or hospital visit with one of the following ‘homeless’ indicators:  DAD   - Missing postal code - Institution Type = Supportive Housing - ICD-10 codes: Z590 or Z591   NACRS   - Missing postal code - ICD-10 codes: Z590 or Z591 - Residence Type = “Homeless” or “Shelter”   OMHRS   - ICD-10 codes: Z590 or Z591 - Prior or usual residential status = ‘homeless’ - Living arrangement at discharge = ‘homeless’ - Admitted from = ‘homeless’ |
| Low-income or disability support public drug plan at index | ODB | If index methadone reimbursed through Ontario drug benefits program, with plan code = ‘C’ or ‘D’ |
| Residence in northern Ontario at index | RPDB | Local Health Integration Network = 13 or 14 |
| Year of Index date | NMS | Index date |
| Charlson score calculated at index | DAD | Validated definition^1^ |
| Any diagnosis for HIV recorded prior to index date | HIV | Validated definition^2^ |
| Any diagnosis for COPD recorded prior to index date | COPD | Validated definition^3^ |
| Any diagnosis for asthma recorded prior to index date | Asthma | Validated definition^4^ |
| Prior diagnosis of chronic kidney disease (5 years prior to index date) | DAD, OHIP | ICD-10 codes: E102, E112, E132, E142, I12, I13, N08, N18, N19  OHIP codes: 403, 585 |
| Prior diagnosis of liver disease  (365 days prior to index date) | DAD, NACRS | ICD-10 codes: K70.0, K70.2, K73.X, K754, K758, K75.9, K76.0, B18.0, B18.1, B18.2, B18.8, B18.9 |
| COPD related hospital or ED visit (365 days prior to index date) | DAD, NACRS | ICD-10 codes: J10-18, J20, J22, J40, J41, J42, J43, or J44 |
| Asthma related hospital or ED visit (365 days prior to index date) | DAD, NACRS | ICD-10 codes: J45.xx |
| Mental health related hospital or ED visit (3 years prior to index date) | DAD, NACRS, OMHRS | Any mental health and addictions (based on main/most responsible diagnoses):  ICD-9 Codes: Any OMHRS record (excluding 290.x, 294.0x-294.7x, 294.9x)  ICD-10 Codes: DX10CODE1= F06-F99 or DX10CODE2-DX10CODE10 = X60-X84, Y10-Y19, Y28 when DX10CODE1 ne F06-F99 |
| Psychotic disorders related outpatient visit (3 years prior to index date) | OHIP | OHIP fee codes: 295, 297, 298, 296 |
| Behavioral and neuro-developmental disorders related outpatient visit (3 years prior to index date) | OHIP | OHIP fee codes: 299, 313, 314, 315 |
| Other mental health disorders related outpatient visit (3 years prior to index date) | OHIP | OHIP fee codes: 301, 302, 306, 307 |
| Healthcare interactions pertaining to alcohol use disorder (3 years prior to index date) | DAD, NACRS, OMHRS | ICD-10 (DAD/NACRS/OMHRS): F10, G62.1; G31.2; G72.1; I42.6; K29.2; K70.0-K70.4; K70.9; K85.2; K86.0  ICD-9 (OMHRS): 291.x [excl. 291.82], 303.x, 305.0 |
| Healthcare interactions pertaining to harmful or dependent stimulant use (3 years prior to index date) | DAD, NACRS, OMHRS | ICD-10: F140- F159, T436, T405 |
| Healthcare interactions pertaining to harmful or dependent sedative-hypnotic use (3 years prior to index date) | DAD, NACRS, OMHRS | ICD-10: F131, F132, T423, T424, T426, T427 |
| Hospital or ED visit for injection-related infection (3 years prior to index date) | DAD, NACRS | ICD-10 codes:   - Osteomyelitis: M86*, M899 - Endocarditis: B376, I33, I34*, I35, I36, I37, I38, I39 - Sepsis: A40, A41, I269, I400, R572, R651, R659 - Skin and soft tissue infection: I80, L97, L988, M793, A480, G06, G09, K630, K650, K750, L02, L03, M5402, M726, N10, R02 |
| Hospital or ED visit for alcohol related toxicity event (365 days prior to index date) | DAD, NARCRS | ICD-10: T510 |
| Hospital or ED visit for benzodiazepine related toxicity event (365 days prior to index date) | DAD, NARCRS | ICD-10: T424 |
| Hospital or ED visit for stimulant related toxicity event (365 days prior to index date) | DAD, NARCRS | ICD-10: T436 |
| Hospital or ED visit for opioid toxicity related toxicity event (365 days prior to index date) | DAD, NACRS | ICD-10: T40.0, T40.1, T40.2, T40.3, T40.4, T40.6 |
| Non-OUD related outpatient visit(s) (365 days prior to index date) | OHIP | Number of OHIP billings overall, excluding billings related to OUD (i.e., K682, K683, K684, A957, K680, G040, G041, G042, G043) |
| ED visit(s) (365 days prior to index date) | NACRS | Number of unique ED visits. All ED visits are considered, regardless of diagnoses or procedures identified. |
| Hospital visit(s) (365 days prior to index date) | DAD | Number of unique hospital visits. All hospital visits are considered, regardless of diagnoses or procedures identified. |
| Attachment of a primary care provider | CHC, ODB, OHIP, PCPOP | Validated definition |
| Prior history of Prescription medications | NMS, ODB | Controlled Prescription Medication Use: A dispense record for a stimulant, benzodiazepine or non-OAT opioid in the 30 days prior to index date.  Direct acting antivirals: Prior dispense record for a direct acting antiviral in the 1 year prior to index date  Opioid Agonist Treatment Use: Prior dispense record for another OAT product (i.e., methadone, buprenorphine/naloxone, SROM, injection buprenorphine, implant buprenorphine) in the 1 year prior to index date.  Immediate release hydromorphone: Prior dispense record for immediate release hydromorphone with strength = 4 or 8mg. |
| **Methadone adherence between initiation and index date related covariates** | | |
| Dispense record for methadone the day immediately prior to index date | NMS | Methadone dispensed the day prior to index date |
| Missed methadone doses between methadone initiation date and index date | NMS | Number of days without a dispense record for methadone in between the date of initiation and index date |
| Methadone dose on treatment initiation date | NMS | Methadone dose dispensed (in mg) on date of initiation (i.e., treatment day 1) |
| OUD-related outpatient visit between methadone initiation and index date | OHIP | Record for any OUD-related outpatient visit.  OHIP codes: K682, K683, K684, A957, K680, G040, G041, G042, G043 |
| Days until a dose increase was provided or date of randomly assigned index date | NMS | Determine whether index date was defined on treatment day 4, 5 or 6. |
| **Prescriber-related covariates** | | |
| Prescriber type | NMS, IPDB | Prescriber identified on the methadone dispense record provided on index date |
| OAT prescriber volume | NMS, IPDB | Defined based on the distribution of unique OAT clients per prescriber identified in our study cohort |
| Years in clinical practice | NMS, IPDB | Year of index date minus medical school graduation year |

**REFERENCES:**

1. Quan H, Li B, Couris CM, et al. Updating and validating the Charlson comorbidity index and score for risk adjustment in hospital discharge abstracts using data from 6 countries. Am J Epidemiol. Mar 15 2011;173(6):676-82. doi:10.1093/aje/kwq433

2. Antoniou T, Zagorski B, Loutfy MR, Strike C, Glazier RH. Validation of case-finding algorithms derived from administrative data for identifying adults living with human immunodeficiency virus infection. PLoS One. 2011;6(6):e21748. doi:10.1371/journal.pone.0021748

3. Gershon AS, Wang C, Guan J, Vasilevska-Ristovska J, Cicutto L, To T. Identifying individuals with physcian diagnosed COPD in health administrative databases. COPD. Oct 2009;6(5):388-94. doi:10.1080/15412550903140865

4. Gershon AS, Wang C, Guan J, Vasilevska-Ristovska J, Cicutto L, To T. Identifying patients with physician-diagnosed asthma in health administrative databases. Can Respir J. 2009;16(6):183-8. doi:10.1155/2009/963098
