# Supplementary material for: Association between early methadone dose titration and treatment discontinuation and opioid toxicity: A retrospective cohort study
Source: PLoS Med. 2026 Apr 9;23(4):e1004748. doi: 10.1371/journal.pmed.1004748 (PMC13065010; doi:10.1371/journal.pmed.1004748)
Supplement: S5 Table — (DOCX) [file pmed.1004748.s005.docx]

**S5 Table.** Crude association between early dose titration and study outcomes

| **Outcome** | **Rate per 100 person-years (95% CI)** | | **Hazard Ratio^a^ (95% CI)** |
| --- | --- | --- | --- |
|  | **Unexposed**  **(No dose increase)** | **Exposed**  **(Dose increase)** |  |
| **Methadone Discontinuation** | 327.9 (317.5 - 338.6) | 189.4 (184.1 - 194.9) | Interval 1^b^: 0.40 (0.37, 0.44) |
|  |  |  | Interval 2^c^: 0.64 (0.59, 0.70) |
|  |  |  | Interval 3^d^: 0.75 (0.69, 0.82) |
|  |  |  | Interval 4^e^: 0.82 (0.73, 0.91) |
|  | | | |
| Intention to treat | 13.6 (12.3 - 15.2) | 9.5 (8.6 - 10.5) | 0.70 (0.60, 0.80) |
| While on treatment | 6.8 (5.4 – 8.5) | 5.2 (4.4 – 6.2) | 0.83 (0.63, 1.11) |
| Methadone toxicity  (while on treatment) | 2.3 (1.6 – 3.4) | 1.8 (1.4 – 2.5) | 0.87 (0.54, 1.43) |
| Non-methadone toxicity  (while on treatment) | 5.1 (3.9 – 6.5) | 3.6 (2.9 – 4.4) | 0.78 (0.56, 1.08) |

**Foot Notes:**

^a^Reference group: Unexposed

^b^0 to 7 days of follow-up

^c^8 to 30 days of follow-up

^d^31 to 90 days of follow-up

^e^91 to 181 days of follow-up

CI, confidence interval
