# Supplementary material for: Association between early methadone dose titration and treatment discontinuation and opioid toxicity: A retrospective cohort study
Source: PLoS Med. 2026 Apr 9;23(4):e1004748. doi: 10.1371/journal.pmed.1004748 (PMC13065010; doi:10.1371/journal.pmed.1004748)
Supplement: S6 Table — (DOCX) [file pmed.1004748.s006.docx]

| **S6 Table. Baseline characteristics of incident methadone recipients in Ontario, Canada, January 1, 2017, to December 31, 2022, comparing no dose increase versus provision of a dose increase <15mg** | | | | | |
| --- | --- | --- | --- | --- | --- |
|  | **Before weighting** | | | **Standardized mean difference** | |
|  | **Unexposed**  **(No dose titration;**  **N=** **5,256^a^)** | | **Exposed**  **(<15mg dose titration;**  **N=5,123^a^)** | **Before weighting** | **After Weighting** |
| **Demographic Characteristics** |  |  | |  |  |
| **Age (Mean, SD)** | 36.0 (9.9) | | 36.9 (10.8) | 0.08 | <0.01 |
| **Female** | 1,818 (34.6%) | | 1,863 (36.4%) | 0.04 | 0.01 |
| **Income Quintile** |  | |  |  |  |
| 1 | 2,058 (39.2%) | | 1,897 (37.0%) | 0.04 | <0.01 |
| 2 | 1,258 (23.9%) | | 1,300 (25.4%) | 0.03 | 0.01 |
| 3 | 929 (17.7%) | | 843 (16.5%) | 0.03 | 0.01 |
| 4 | 604 (11.5%) | | 638 (12.5%) | 0.03 | 0.01 |
| 5 | 407 (7.7%) | | 445 (8.7%) | 0.03 | <0.01 |
| **Hospital flagged homelessness** (1 year prior) | 475 (9.0%) | | 303 (5.9%) | 0.12 | 0.01 |
| **Low-income or disability support public drug plan** | 2,695 (51.3%) | | 2,608 (50.9%) | 0.01 | <0.01 |
| **Residence in northern Ontario** | 589 (11.2%) | | 351 (6.9%) | 0.15 | 0.01 |
| **Urban location of residence** | 4,721 (89.8%) | | 4,619 (90.2%) | 0.01 | 0.02 |
| **Year of index date** |  | |  |  |  |
| 2017 | 1,098 (20.9%) | | 1,456 (28.4%) | 0.18 | 0.01 |
| 2018 | 881 (16.8%) | | 1,098 (21.4%) | 0.12 | <0.01 |
| 2019 | 877 (16.7%) | | 883 (17.2%) | 0.01 | <0.01 |
| 2020 | 883 (16.8%) | | 825 (16.1%) | 0.02 | 0.01 |
| 2021 | 836 (15.9%) | | 541 (10.6%) | 0.16 | <0.01 |
| 2022 | 676 (12.9%) | | 318 (6.2%) | 0.23 | <0.01 |
| 2023 | <=5 (0.1%) | | <=5 (0.0%) | 0.02 | 0.01 |
| **Comorbidities** |  |  | |  |  |
| **Charlson score** |  | |  |  |  |
| No hospital visits | 3,580 (68.1%) | | 3,409 (66.5%) | 0.03 | 0.01 |
| 0 | 1,386 (26.4%) | | 1,420 (27.7%) | 0.03 | 0.00 |
| 1 | 202 (3.8%) | | 193 (3.8%) | 0.00 | 0.02 |
| 2+ | 88 (1.7%) | | 101 (2.0%) | 0.02 | 0.00 |
| **Human Immunodeficiency Virus** | 45 (0.9%) | | 39 (0.8%) | 0.01 | 0.01 |
| **COPD** | 346 (6.6%) | | 467 (9.1%) | 0.09 | 0.00 |
| **Asthma** | 1,295 (24.6%) | | 1,341 (26.2%) | 0.04 | 0.01 |
| **Chronic Kidney Disease** (5 years prior) | 44 (0.8%) | | 65 (1.3%) | 0.04 | 0.01 |
| **Liver Disease** (1 year prior) | 73 (1.4%) | | 68 (1.3%) | 0.01 | 0.01 |
| **COPD related hospital or ED visit**  (1 year prior) | 142 (2.7%) | | 140 (2.7%) | 0.00 | 0.00 |
| **Asthma related hospital or ED visit**  (1 year prior) | 35 (0.7%) | | 32 (0.6%) | 0.01 | 0.01 |
| **Mental health related hospital or ED visit**  (3 years prior) | 4,301 (81.8%) | | 4,155 (81.1%) | 0.02 | 0.00 |
| **Psychotic disorders related outpatient visit**  (3 years prior) | 773 (14.7%) | | 706 (13.8%) | 0.03 | 0.00 |
| **Behavioral and neuro-developmental disorders related outpatient visit**  (3 years prior) | 290 (5.5%) | | 261 (5.1%) | 0.02 | 0.01 |
| **Other mental health disorders related outpatient visit** (3 years prior) | 867 (16.5%) | | 784 (15.3%) | 0.03 | 0.01 |
| **Alcohol use disorder** (3 years prior) | 466 (8.9%) | | 436 (8.5%) | 0.01 | 0.00 |
| **Stimulant harmful use or dependence**  (3 years prior) | 811 (15.4%) | | 601 (11.7%) | 0.11 | 0.01 |
| **Sedative-hypnotic harmful use or dependence**  (3 years prior) | 163 (3.1%) | | 152 (3.0%) | 0.01 | 0.01 |
| **Hospital or ED visit for injection-related infection** (3 years prior) | 1,170 (22.3%) | | 932 (18.2%) | 0.10 | 0.01 |
| **Hospital or ED visit for toxicity (1 year prior)** |  | | |  | |
| **Alcohol-related** | 19 (0.4%) | | 15 (0.3%) | 0.01 | 0.00 |
| **Benzodiazepine-related** | 56 (1.1%) | | 44 (0.9%) | 0.02 | 0.01 |
| **Stimulant-related toxicity** | 56 (1.1%) | | 36 (0.7%) | 0.04 | 0.00 |
| **Non-fatal opioid toxicity** | 567 (10.8%) | | 417 (8.1%) | 0.09 | 0.01 |
| **Healthcare utilization (1 year prior)** |  | | |  | |
| **Non-OUD related outpatient visits** | 4,543 (86.4%) | | 4,589 (89.6%) |  |  |
| Mean (SD) | 7.6 (10.5) | | 8.2 (11.4) | 0.06 | <0.01 |
| 0 | 713 (13.6%) | | 534 (10.4%) |  |  |
| 1-4 | 2,153 (41.0%) | | 2,078 (40.6%) |  |  |
| 5-10 | 1,197 (22.8%) | | 1,205 (23.5%) |  |  |
| 11+ | 1,193 (22.7%) | | 1,306 (25.5%) |  |  |
| **ED visits** | 3,061 (58.2%) | | 2,970 (58.0%) |  |  |
| Mean (SD) | 2.1 (4.1) | | 2.0 (4.4) | 0.01 | 0.01 |
| 0 | 2,195 (41.8%) | | 2,153 (42.0%) |  |  |
| 1 | 1,065 (20.3%) | | 1,073 (20.9%) |  |  |
| 2-3 | 1,047 (19.9%) | | 1,016 (19.8%) |  |  |
| 4+ | 949 (18.1%) | | 881 (17.2%) |  |  |
| **Hospital visit** | 599 (11.4%) | | 552 (10.8%) |  |  |
| Mean (SD) | 0.2 (0.6) | | 0.2 (0.6) | 0.02 | <0.01 |
| 0 | 4,657 (88.6%) | | 4,571 (89.2%) |  |  |
| 1+ | 599 (11.4%) | | 552 (10.8%) |  |  |
| **Attachment to Primary Care** | 4,725 (89.9%) | | 4,729 (92.3%) | 0.08 | <0.01 |
| **Medication Use History** |  |  | |  |  |
| **Controlled Prescription Medication Use**  (30 days prior) | 540 (10.3%) | | 778 (15.2%) | 0.15 | 0.02 |
| Stimulants | 181 (3.4%) | | 174 (3.4%) | 0 | 0.02 |
| Benzodiazepines | 427 (8.1%) | | 674 (13.2%) | 0.16 | 0.01 |
| non-OAT opioids | 0 (0.0%) | | 0 (0.0%) |  |  |
| **Direct acting antivirals** (1 year prior) | 87 (1.7%) | | 63 (1.2%) | 0.04 | 0.00 |
| **Opioid Agonist Treatment Use** (1 year prior) | 2,647 (50.4%) | | 1,792 (35.0%) | 0.31 | 0.02 |
| Methadone | 2,105 (40.0%) | | 1,202 (23.5%) | 0.36 | 0.01 |
| Buprenorphine/naloxone | 956 (18.2%) | | 736 (14.4%) | 0.1 | 0.00 |
| Long-acting buprenorphine | 12 (0.2%) | | 10 (0.2%) | 0.01 | 0.00 |
| Slow-Release Oral Morphine | 83 (1.6%) | | 45 (0.9%) | 0.06 | 0.01 |
| **Immediate release hydromorphone**  (1 year prior) | 115 (2.2%) | | 166 (3.2%) | 0.06 | 0.01 |
| **Methadone adherence characteristics following treatment initiation** | | | |  | |
| **Dispense record for methadone the day before index date** | 4,248 (80.8%) | | 4,714 (92.0%) | 0.33 | <0.01 |
| **Missed methadone doses between methadone initiation date and index date** |  | |  |  |  |
| 0 | 3,460 (65.8%) | | 4,444 (86.7%) | 0.51 | 0.01 |
| 1 | 1,325 (25.2%) | | 507 (9.9%) | 0.41 | 0.01 |
| 2 | 471 (9.0%) | | 172 (3.4%) | 0.23 | 0.00 |
| **Methadone dose on treatment initiation date** |  | |  |  |  |
| Median (IQR) | 25 (20-30) | | 20 (20-25) |  |  |
| Mean (SD) | 25.3 (7.8) | | 22.1 (6.0) | 0.48 | <0.01 |
| **Methadone dose on index date,** |  | |  |  |  |
| Median (IQR) | 25 (20-30) | | 30 (30-35) |  |  |
| Mean (SD) | 25.3 (7.8) | | 31.2 (6.4) |  |  |
| **OUD-related outpatient visit between methadone initiation and index date** | 561 (10.7%) | | 1,497 (29.2%) | 0.48 | 0.01 |
| **Physician Characteristics at Index** |  | | |  | |
| **Physician Specialty – Family Practitioner** | 4,096 (77.9%) | | 4,163 (81.3%) | 0.08 | 0.01 |
| **Prescriber OAT Client Volume** |  | | |  | |
| Low (lowest 50th percentile) | 614 (11.7%) | | 471 (9.2%) | 0.08 | 0.02 |
| Moderate (51st to 80th percentile) | 1,616 (30.7%) | | 1,676 (32.7%) | 0.04 | 0.00 |
| High (top 20th percentile) | 3,026 (57.6%) | | 2,976 (58.1%) | 0.01 | 0.01 |
| **Years in Clinical Practice** |  | |  |  |  |
| Mean (SD) | 23.4 (10.9) | | 25.8 (10.6) | 0.23 | <0.01 |
| <10 years | 641 (12.2%) | | 386 (7.5%) | 0.16 |  |
| 10-19 years | 1,372 (26.1%) | | 1,038 (20.3%) | 0.14 |  |
| 20+ years | 3,243 (61.7%) | | 3,699 (72.2%) | 0.22 |  |

Footnotes:

^a^ Derived after applying propensity score trimming to the study cohort

SD, standard deviation; ED, emergency department; OUD, opioid use disorder; OAT, opioid agonist treatment; COPD, chronic obstructive pulmonary disease; IQR, interquartile range
