# Supplementary material for: Association between early methadone dose titration and treatment discontinuation and opioid toxicity: A retrospective cohort study
Source: PLoS Med. 2026 Apr 9;23(4):e1004748. doi: 10.1371/journal.pmed.1004748 (PMC13065010; doi:10.1371/journal.pmed.1004748)
Supplement: S7 Table — (DOCX) [file pmed.1004748.s007.docx]

| **S7 Table. Baseline characteristics of incident methadone recipients in Ontario, Canada, January 1, 2017, to December 31, 2022, comparing no dose increase versus provision of a dose increase ≥15mg** | | | | | |
| --- | --- | --- | --- | --- | --- |
|  | **Before weighting** | | | **Standardized mean difference** | |
|  | **Unexposed**  **(No dose titration;**  **N=5,271^a^)** | | **Exposed**  **(≥15mg dose titration;**  **N=3,173^a^)** | **Before weighting** | **After Weighting** |
| **Demographic Characteristics** |  |  | |  |  |
| **Age (Mean, SD)** | 36.1 (9.9) | | 36.5 (10.0) | 0.05 | <0.01 |
| **Female** | 1,831 (34.7%) | | 1,056 (33.3%) | 0.03 | <0.01 |
| **Income Quintile** |  | |  |  |  |
| 1 | 2,068 (39.2%) | | 1,215 (38.3%) | 0.02 | 0.01 |
| 2 | 1,258 (23.9%) | | 728 (22.9%) | 0.02 | 0.00 |
| 3 | 930 (17.6%) | | 550 (17.3%) | 0.01 | 0.00 |
| 4 | 608 (11.5%) | | 401 (12.6%) | 0.03 | 0.01 |
| 5 | 407 (7.7%) | | 279 (8.8%) | 0.04 | 0.01 |
| **Hospital flagged homelessness** (1 year prior) | 483 (9.2%) | | 240 (7.6%) | 0.06 | 0.02 |
| **Low-income or disability support public drug plan** | 2,713 (51.5%) | | 1,623 (51.2%) | 0.01 | 0.02 |
| **Residence in northern Ontario** | 597 (11.3%) | | 458 (14.4%) | 0.09 | 0.01 |
| **Urban location of residence** | 4,733 (89.8%) | | 2,823 (89.0%) | 0.03 | 0.02 |
| **Year of index date** |  | |  |  |  |
| 2017 | 1,079 (20.5%) | | 515 (16.2%) | 0.11 | 0.01 |
| 2018 | 860 (16.3%) | | 386 (12.2%) | 0.12 | 0.01 |
| 2019 | 880 (16.7%) | | 488 (15.4%) | 0.04 | 0.01 |
| 2020 | 889 (16.9%) | | 607 (19.1%) | 0.06 | 0.00 |
| 2021 | 852 (16.2%) | | 676 (21.3%) | 0.13 | 0.00 |
| 2022 | 705 – 710 | | 495 - 500 | 0.07 | 0.02 |
| 2023 | <=5 (0.1%) | | <=5 (0.1%) | 0.01 | 0.03 |
| **Comorbidities** |  |  | |  |  |
| **Charlson score** |  | |  |  |  |
| No hospital visits | 3,574 (67.8%) | | 2,162 (68.1%) | 0.01 | 0.00 |
| 0 | 1,401 (26.6%) | | 830 (26.2%) | 0.01 | 0.01 |
| 1 | 209 (4.0%) | | 122 (3.8%) | 0.01 | 0.01 |
| 2+ | 87 (1.7%) | | 59 (1.9%) | 0.02 | 0.00 |
| **Human Immunodeficiency Virus** | 43 (0.8%) | | 25 (0.8%) | 0.00 | 0.01 |
| **COPD** | 347 (6.6%) | | 230 (7.2%) | 0.03 | 0.00 |
| **Asthma** | 1,288 (24.4%) | | 777 (24.5%) | 0.00 | 0.01 |
| **Chronic Kidney Disease** (5 years prior) | 44 (0.8%) | | 28 (0.9%) | 0.01 | 0.01 |
| **Liver Disease** (1 year prior) | 74 (1.4%) | | 40 (1.3%) | 0.01 | 0.02 |
| **COPD related hospital or ED visit**  (1 year prior) | 136 (2.6%) | | 67 (2.1%) | 0.03 | 0.01 |
| **Asthma related hospital or ED visit**  (1 year prior) | 34 (0.6%) | | 21 (0.7%) | 0.00 | 0.00 |
| **Mental health related hospital or ED visit**  (3 years prior) | 4,322 (82.0%) | | 2,590 (81.6%) | 0.01 | 0.01 |
| **Psychotic disorders related outpatient visit**  (3 years prior) | 773 (14.7%) | | 364 (11.5%) | 0.09 | 0.00 |
| **Behavioral and neuro-developmental disorders related outpatient visit**  (3 years prior) | 290 (5.5%) | | 168 (5.3%) | 0.01 | 0.01 |
| **Other mental health disorders related outpatient visit** (3 years prior) | 866 (16.4%) | | 476 (15.0%) | 0.04 | 0.00 |
| **Alcohol use disorder** (3 years prior) | 467 (8.9%) | | 273 (8.6%) | 0.01 | 0.00 |
| **Stimulant harmful use or dependence**  (3 years prior) | 811 (15.4%) | | 419 (13.2%) | 0.06 | 0.00 |
| **Sedative-hypnotic harmful use or dependence**  (3 years prior) | 168 (3.2%) | | 87 (2.7%) | 0.03 | 0.00 |
| **Hospital or ED visit for injection-related infection** (3 years prior) | 1,192 (22.6%) | | 662 (20.9%) | 0.04 | 0.01 |
| **Hospital or ED visit for toxicity (1 year prior)** |  | | |  | |
| **Alcohol-related** | 20 (0.4%) | | 14 (0.4%) | 0.01 | 0.01 |
| **Benzodiazepine-related** | 55 (1.0%) | | 25 (0.8%) | 0.03 | <0.01 |
| **Stimulant-related toxicity** | 57 (1.1%) | | 40 (1.3%) | 0.02 | 0.01 |
| **Non-fatal opioid toxicity** | 576 (10.9%) | | 306 (9.6%) | 0.04 | 0.01 |
| **Healthcare utilization (1 year prior)** |  | | |  | |
| **Non-OUD related outpatient visits** | 4,553 (86.4%) | | 2,727 (85.9%) |  |  |
| Mean (SD) | 7.5 (10.6) | | 7.1 (9.4) | 0.05 | 0.02 |
| 0 | 718 (13.6%) | | 446 (14.1%) |  |  |
| 1-4 | 2,163 (41.0%) | | 1,312 (41.3%) |  |  |
| 5-10 | 1,205 (22.9%) | | 730 (23.0%) |  |  |
| 11+ | 1,185 (22.5%) | | 685 (21.6%) |  |  |
| **ED visits** | 3,077 (58.4%) | | 1,852 (58.4%) |  |  |
| Mean (SD) | 2.1 (3.9) | | 1.9 (3.4) | 0.04 | <0.01 |
| 0 | 2,194 (41.6%) | | 1,321 (41.6%) |  |  |
| 1 | 1,075 (20.4%) | | 685 (21.6%) |  |  |
| 2-3 | 1,048 (19.9%) | | 630 (19.9%) |  |  |
| 4+ | 954 (18.1%) | | 537 (16.9%) |  |  |
| **Hospital visit** | 604 (11.5%) | | 359 (11.3%) |  |  |
| Mean (SD) | 0.2 (0.6) | | 0.2 (0.6) | 0.01 | 0.01 |
| 0 | 4,667 (88.5%) | | 2,814 (88.7%) |  |  |
| 1+ | 604 (11.5%) | | 359 (11.3%) |  |  |
| **Attachment to Primary Care** | 4,731 (89.8%) | | 2,820 (88.9%) | 0.03 | <0.01 |
| **Medication Use History** |  |  | |  |  |
| **Controlled Prescription Medication Use**  (30 days prior) | 538 (10.2%) | | 290 (9.1%) | 0.04 | 0.00 |
| Stimulants | 183 (3.5%) | | 107 (3.4%) | 0.01 | 0.00 |
| Benzodiazepines | 422 (8.0%) | | 211 (6.6%) | 0.05 | 0.00 |
| non-OAT opioids |  | |  |  |  |
| **Direct acting antivirals** (1 year prior) | 92 (1.7%) | | 46 (1.4%) | 0.02 | 0.00 |
| **Opioid Agonist Treatment Use** (1 year prior) | 2,667 (50.6%) | | 1,439 (45.4%) | 0.11 | 0.01 |
| Methadone | 2,123 (40.3%) | | 1,023 (32.2%) | 0.17 | 0.01 |
| Buprenorphine/naloxone | 969 (18.4%) | | 627 (19.8%) | 0.04 | 0.00 |
| Long-acting buprenorphine | 12 (0.2%) | | 8 (0.3%) | 0.01 | 0.00 |
| Slow-Release Oral Morphine | 93 (1.8%) | | 49 (1.5%) | 0.02 | 0.01 |
| **Immediate release hydromorphone**  (1 year prior) | 117 (2.2%) | | 94 (3.0%) | 0.05 | 0.00 |
| **Methadone adherence characteristics following treatment initiation** | | | |  | |
| **Dispense record for methadone the day before index date** | 4,255 (80.7%) | | 2,930 (92.3%) | 0.35 | <0.01 |
| **Missed methadone doses between methadone initiation date and index date** |  | |  |  |  |
| 0 | 3,462 (65.7%) | | 2,756 (86.9%) | 0.51 | 0.01 |
| 1 | 1,332 (25.3%) | | 320 (10.1%) | 0.41 | 0.01 |
| 2 | 477 (9.0%) | | 97 (3.1%) | 0.25 | 0.00 |
| **Methadone dose on treatment initiation date** |  | |  |  |  |
| Median (IQR) | 30 (20-30) | | 30 (20-30) |  |  |
| Mean (SD) | 25.6 (8.0) | | 26.8 (5.9) | 0.17 | 0.01 |
| **Methadone dose on index date,** |  | |  |  |  |
| Median (IQR) | 30 (20-30) | | 45 (35-45) |  |  |
| Mean (SD) | 25.6 (8.0) | | 46.5 (61.6) |  |  |
| **OUD-related outpatient visit between methadone initiation and index date** | 560 (10.6%) | | 836 (26.3%) | 0.41 | 0.01 |
| **Physician Characteristics at Index** |  | | |  | |
| **Physician Specialty -**  **Family Practitioner** | 4,091 (77.6%) | | 2,419 (76.2%) | 0.03 | 0.04 |
| **Prescriber OAT Client Volume** |  | | |  | |
| Low (lowest 50th percentile) | 614 (11.6%) | | 264 (8.3%) | 0.11 | 0.02 |
| Moderate (51st to 80th percentile) | 1,638 (31.1%) | | 1,087 (34.3%) | 0.07 | 0.02 |
| High (top 20th percentile) | 3,019 (57.3%) | | 1,822 (57.4%) | 0.00 | 0.00 |
| **Years in Clinical Practice** |  | |  |  |  |
| Mean (SD) | 23.3 (10.9) | | 23.3 (10.5) | 0.00 | 0.01 |
| <10 years | 645 (12.2%) | | 353 (11.1%) | 0.03 |  |
| 10-19 years | 1,382 (26.2%) | | 779 (24.6%) | 0.04 |  |
| 20+ years | 3,244 (61.5%) | | 2,041 (64.3%) | 0.06 |  |

Footnotes:

^a^ Derived after applying propensity score trimming to the study cohort

SD, standard deviation; ED, emergency department; OUD, opioid use disorder; OAT, opioid agonist treatment; COPD, chronic obstructive pulmonary disease; IQR, interquartile range
