# Supplementary material for: Association between early methadone dose titration and treatment discontinuation and opioid toxicity: A retrospective cohort study
Source: PLoS Med. 2026 Apr 9;23(4):e1004748. doi: 10.1371/journal.pmed.1004748 (PMC13065010; doi:10.1371/journal.pmed.1004748)
Supplement: S8 Table — (DOCX) [file pmed.1004748.s008.docx]

**S8 Table.** Association between early dose titration and study outcomes, stratified analysis

| **Outcome** | **Rate^a^ per 100 person-years (95% CI)** | | **Hazard Ratio^a,b^ (95% CI)** |
| --- | --- | --- | --- |
|  | **Unexposed**  **(No dose increase)** | **Exposed**  **(Dose increase)** |  |
| **Exposed group restricted to those with a dose increase of <15mg on index** | | | |
| **Methadone Discontinuation** | 280.4 (267.1 - 294.2) | 205.3 (195.5 - 215.6) | Interval 1^c^: 0.55 (0.50, 0.61) |
|  |  |  | Interval 2^d^: 0.82 (0.75, 0.90) |
|  |  |  | Interval 3^e^: 0.83 (0.76, 0.91) |
|  |  |  | Interval 4^f^: 0.91 (0.82, 1.02) |
| **Opioid toxicity** | | | |
| Intention to treat | 11.9 (10.5 - 13.4) | 8.4 (7.1 - 9.9) | 0.71 (0.57, 0.87) |
| While on treatment | 6.67 (5.18-8.60) | 4.51 (3.52-5.78) | 0.71 (0.50, 1.01) |
| Methadone toxicity  (while on treatment) | 2.68 (1.68-4.28) | 1.67 (1.16-2.40) | 0.66 (0.37, 1.18) |
| Non-methadone toxicity  (while on treatment) | 3.9 (3.00-5.30) | 2.84 (2.04-3.95) | 0.75 (0.49, 1.15) |
| **Exposed group restricted to those with a dose increase of ≥15mg on index** | | | |
| **Methadone Discontinuation** | 314.6 (301.2 - 328.6) | 235.3 (221.7 - 249.8) | Interval 1^c^: 0.57 (0.51, 0.64) |
|  |  |  | Interval 2^d^: 0.74 (0.67, 0.82) |
|  |  |  | Interval 3^e^: 0.94 (0.85, 1.04) |
|  |  |  | Interval 4^f^: 0.96 (0.84, 1.09) |
| **Opioid toxicity** | | | |
| Intention to treat | 13.3 (11.9 - 14.9) | 12.5 (10.5 - 14.9) | 0.95 (0.77, 1.16) |
| While on treatment | 7.22 (5.75-9.06) | 8.08 (5.97-10.93) | Interval 1^c^: 1.07 (0.59, 1.91) |
|  |  |  | Interval 2^d^: 0.97 (0.50, 1.82) |
|  |  |  | Interval 3^e^: 0.86 (0.42, 1.70) |
|  |  |  | Interval 4^f^: 2.23 (1.14, 4.51) |
| Methadone toxicity  (while on treatment) | 2.28 (1.53-3.40) | 1.89 (1.15-3.11) | 0.88 (0.46, 1.66) |
| Non-methadone toxicity  (while on treatment) | 4.94 (3.74-6.51) | 6.19 (4.30-8.91) | Interval 1^c^: 1.17 (0.55, 2.40) |
|  |  |  | Interval 2^d^: 1.05 (0.52, 2.09) |
|  |  |  | Interval 3^e^: 0.74 (0.30, 1.73) |
|  |  |  | Interval 4^f^: 3.29 (1.48, 8.07) |

**Foot Notes:**

^a^stabilised inverse probability treatment weighting

^b^Reference group: Unexposed

^c^0 to 7 days of follow-up

^d^8 to 30 days of follow-up

^e^31 to 90 days of follow-up

^f^91 to 181 days of follow-up

CI, confidence interval
