# Supplementary material for: Association between early methadone dose titration and treatment discontinuation and opioid toxicity: A retrospective cohort study
Source: PLoS Med. 2026 Apr 9;23(4):e1004748. doi: 10.1371/journal.pmed.1004748 (PMC13065010; doi:10.1371/journal.pmed.1004748)
Supplement: S9 Table — (DOCX) [file pmed.1004748.s009.docx]

**S9 Table.** Association between early dose titration and study outcomes, sensitivity analysis

| **Outcome** | **Rate^a^ per 100 person-years (95% CI)** | | **Hazard Ratio^b^ (95% CI)** |
| --- | --- | --- | --- |
|  | **Unexposed**  **(No dose increase)** | **Exposed**  **(Dose increase)** |  |
| **Discontinuation defined as gap of 14 days or more** | | | |
| **Methadone Discontinuation** | 206.96 (197.08-217.34) | 152.03 (146.59-157.67) | Interval 1^c^: 0.49 (0.44, 0.54) |
|  |  |  | Interval 2^d^: 0.79 (0.72, 0.87) |
|  |  |  | Interval 3^e^: 0.86 (0.79, 0.94) |
|  |  |  | Interval 4^f^: 0.89 (0.80, 0.99) |
| **Cohort restricted to those with no missed doses** | | | |
| **Methadone Discontinuation** | 248.74 (234.29-264.07) | 182.50 (176.07-189.16) | Interval 1^c^: 0.57 (0.51, 0.63) |
|  |  |  | Interval 2^d^: 0.81 (0.74, 0.89) |
|  |  |  | Interval 3^e^: 0.82 (0.74, 0.90) |
|  |  |  | Interval 4^f^: 0.83 (0.74, 0.94) |
| **Opioid toxicity (Intention to treat)** | 11.11 (9.32-13.25) | 9.10 (8.12-10.20) | 0.82 (0.66, 1.01) |
| **Cohort restricted to those with index-date = 4** | | | |
| **Methadone Discontinuation** | 310.05 (289.54-332.02) | 214.12 (203.95-224.80) | Interval 1^c^: 0.58 (0.52, 0.64) |
|  |  |  | Interval 2^d^: 0.76 (0.69, 0.84) |
|  |  |  | Interval 3^e^: 0.79 (0.71, 0.88) |
|  |  |  | Interval 4^f^: 0.85 (0.74, 0.97) |
| **Opioid toxicity (Intention to treat)** | 12.61 (10.40-15.30) | 11.47 (9.82-13.40) | 0.91 (0.71, 1.16) |

**Foot Notes:**

^a^stabilised inverse probability treatment weighting

^b^Reference group: Unexposed

^c^0 to 7 days of follow-up

^d^8 to 30 days of follow-up

^e^31 to 90 days of follow-up

^f^91 to 181 days of follow-up

CI, confidence interval
