# Supplementary material for: Association between early methadone dose titration and treatment discontinuation and opioid toxicity: A retrospective cohort study
Source: PLoS Med. 2026 Apr 9;23(4):e1004748. doi: 10.1371/journal.pmed.1004748 (PMC13065010; doi:10.1371/journal.pmed.1004748)
Supplement: S10 Table — (DOCX) [file pmed.1004748.s010.docx]

**S10 Table.** Association between early dose titration and study outcomes, exploratory analysis^a^

| **Outcome** | **Rate^b^ per 100 person-years (95% CI)** | | **Hazard Ratio^b,c^ (95% CI)** |
| --- | --- | --- | --- |
|  | **Unexposed**  **(No dose increase)** | **Exposed**  **(Dose increase)** |  |
| **Methadone Discontinuation** | 289.59  (276.0, 303.9) | 207.0  (200.1, 214.2) | Interval 1^d^: 0.53 (0.48, 0.57) |
|  |  |  | Interval 2^e^: 0.80 (0.74, 0.87) |
|  |  |  | Interval 3^f^: 0.83 (0.76, 0.90) |
|  |  |  | Interval 4^g^: 0.87 (0.78, 0.96) |
| **Opioid toxicity** | | | |
| Intention to treat | 11.0 (9.6, 12.5) | 9.7 (8.7, 10.9) | 0.89 (0.75, 1.05) |
| While on treatment | 7.5 (6.0, 9.2) | 5.5 (4.7, 6.5) | 0.81 (0.62, 1.06) |
| Methadone toxicity  (while on treatment) | 2.4 (1.7, 3.5) | 2.0 (1.5, 2.7) | 0.92 (0.58, 1.45) |
| Non-methadone toxicity  (while on treatment) | 6.7 (5.2, 8.6) | 5.9 (5.0, 7.1) | 0.93 (0.69, 1.26) |

**Foot Notes:**

^a^Study cohort includes first incident use period identified per person over the accrual period

^b^stabilised inverse probability treatment weighting

^c^Reference group: Unexposed

^d^0 to 7 days of follow-up

^e^8 to 30 days of follow-up

^f^31 to 90 days of follow-up

^g^91 to 181 days of follow-up

CI, confidence interval
