# Supplementary material for: Association between early methadone dose titration and treatment discontinuation and opioid toxicity: A retrospective cohort study
Source: PLoS Med. 2026 Apr 9;23(4):e1004748. doi: 10.1371/journal.pmed.1004748 (PMC13065010; doi:10.1371/journal.pmed.1004748)
Supplement: S11 Table — (DOCX) [file pmed.1004748.s011.docx]

**S11 Table.** Characteristics of dose titration following index date (exploratory analysis)

|  | **Overall^a^**  **(N=13,836)** | **Unexposed**  **(N=5,369)** | **Exposed** | | |
| --- | --- | --- | --- | --- | --- |
|  |  |  | **Overall**  **(N=8,635)** | **Dose increase <15mg (N=5,220)** | **Dose increase ≥15mg (N=3,247)** |
| Provision of dose increase within 14 days of index | 9,428 (68.1%) | 2,922 (54.4%) | 6,506 (76.8%) | 3,988 (76.4%) | 2,518 (77.5%) |
| Time to dose increase (median, IQR) | 4 (3-6) | 4 (3-6) | 4 (3-6) | 4 (3-6) | 4 (3-6) |
| Median dose among those titrated (median, IQR) | 40 (35-50) | 35 (30-45) | 45 (40-55) | 40 (35-45) | 60 (50-60) |

Footnotes:

^a^ Exploratory analysis conducted using the full cohort, without application of propensity score trimming

IQR, interquartile range
