# Supplementary figures and images for: Association between early methadone dose titration and treatment discontinuation and opioid toxicity: A retrospective cohort study
Source: PLoS Med. 2026 Apr 9;23(4):e1004748. doi: 10.1371/journal.pmed.1004748 (PMC13065010; doi:10.1371/journal.pmed.1004748)

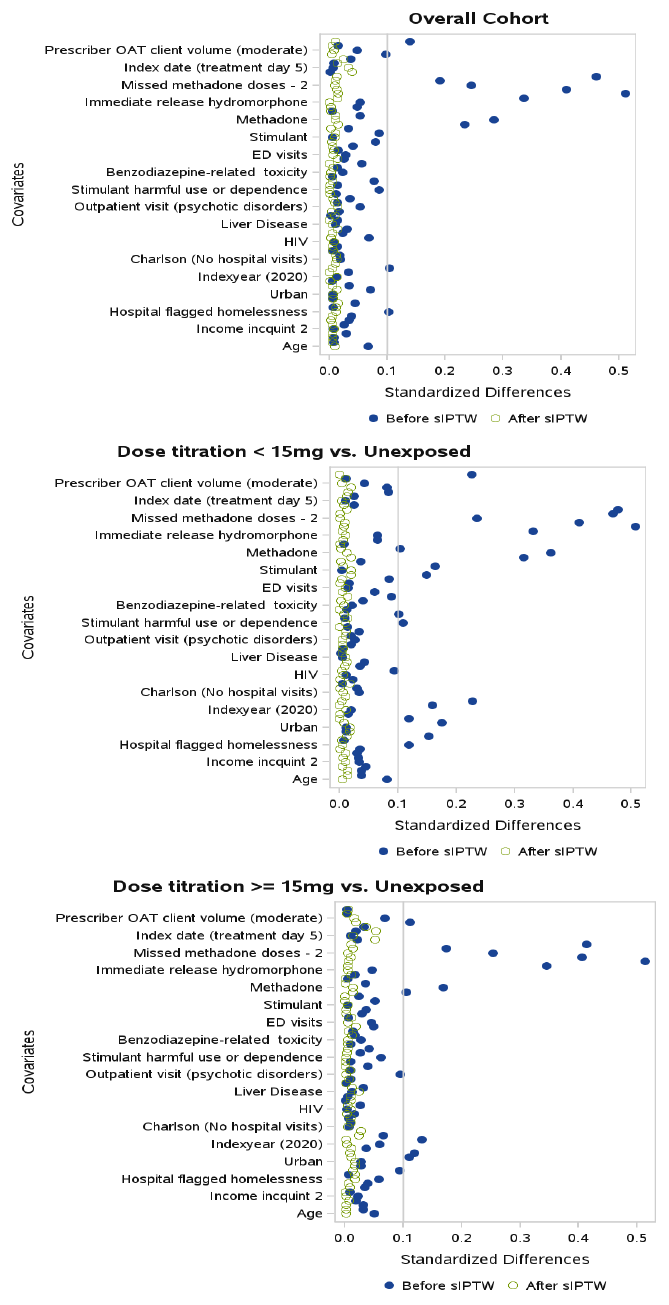

Supplement: S1 Fig — (TIFF) [file pmed.1004748.s012.tiff]

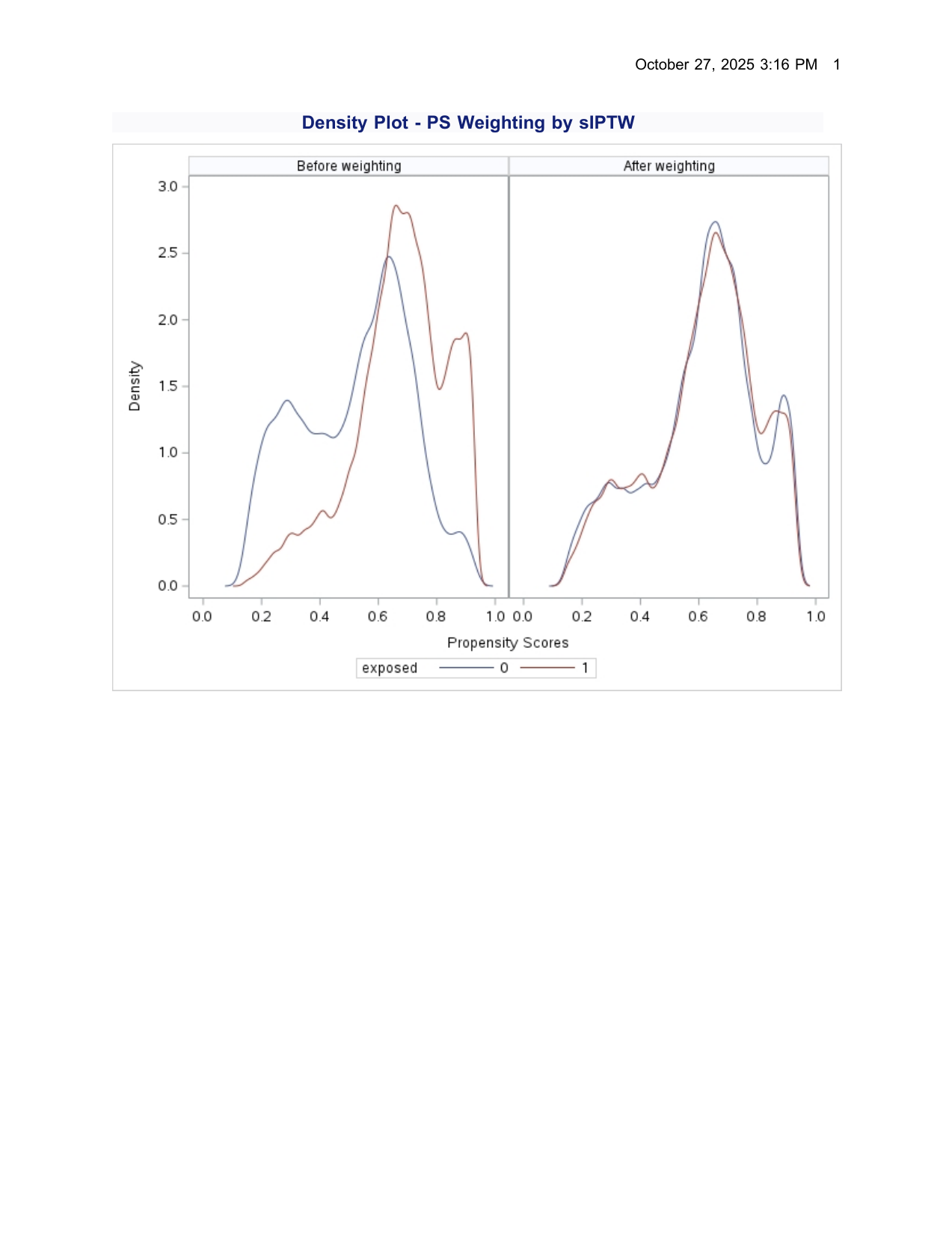

Supplement: S2 Fig — (TIFF) [file pmed.1004748.s013.tiff]
