# Supplementary material for: Association between early methadone dose titration and treatment discontinuation and opioid toxicity: A retrospective cohort study
Source: PLoS Med. 2026 Apr 9;23(4):e1004748. doi: 10.1371/journal.pmed.1004748 (PMC13065010; doi:10.1371/journal.pmed.1004748)
Supplement: Attachment — Submitted filename: early dose titration_reviewer response letter_2025Nov03.doc [file pmed.1004748.s016.doc]

**Comments from the academic editor:**

**This is a really interesting manuscript on an important topic for the North American context and must be appropriately framed in that regard. I think a much more global overview and contextualisation of the paper in the introduction and then discussion is necessary given that this is not an issue for all countries and contexts and the authors must be seen to be (and be) clear about that in the paper - this is an issue where highly potent opioids and likely very high opioid tolerance are the norm among illicit opioid users.**

Response: We thank the editor for their thoughtful review or our manuscript and agree that contextualization of our study within the landscape of Canada’s unregulated drugs supply and how our study findings may be transferable to other regions with a highly potent drug supply is required. We have updated our manuscript accordingly. Please see revised text below.

Revised Text

Abstract: *Clinical guidance in Canada and the United States recommends faster methadone dose titration for individuals using fentanyl.*

Introduction:

*While opioid agonist treatments (OAT) are safe and effective for people with OUD, treatment retention in the United States and Canada remains challenging as majority discontinue in the first few months. This trend may reflect a mismatch between the high opioid tolerance of individuals exposed to a fentanyl-dominated unregulated drug supply and traditional OAT prescribing practices. New clinical guidance recognizes that people who use fentanyl require significantly higher methadone doses than were needed when the unregulated drug supply primarily consisted of heroin. For example, a focus group with people who use fentanyl reported that a minimum methadone dose of 120 mg was needed for adequate suppression of opioid cravings and withdrawal symptoms. Due to requirements for higher methadone doses, evolving clinical guidance in the US and Canada recommends rapid dose titration to reduce time spent*

*receiving a subtherapeutic dose and improve treatment retention.*

Discussion:

*A key finding of this study was that provision of an early dose titration (i.e., during the first four to six days of treatment) supported methadone retention without increased hazard of opioid toxicity among a cohort of individuals using a fentanyl-dominated unregulated drug supply. The safety and feasibility of rapid methadone titration is further supported by emerging evidence from US outpatient opioid treatment programs, with patients reaching doses up to 70 mg within one week of treatment without significant reports of toxicity and improved 30-day methadone retention. Methadone is initiated at subtherapeutic doses due to its highly variable half-life and narrow therapeutic index, which places methadone naïve individuals at risk of bioaccumulation and toxicity. Currently, no clinical tools exist to determine the risk of an iatrogenic methadone toxicity at initiation, necessitating cautious titration. However, individuals who use high potency opioids - such as those in Canada and the US – have a high tolerance for opioids and are likely partially cross-tolerant to methadone, which may permit higher initiation doses and more rapid titration. This is in agreement with our study results, conducted within a region where fentanyl dominates the unregulated drug supply, as early methadone dose titration remained safe even when doses were titrated on treatment day 4 or by 15mg with a median initiation dose of 30mg.*

Conclusion: *Logistical barriers that prevent people with high opioid tolerance from receiving timely dose titration, despite adequate adherence to methadone, should be addressed to improve treatment retention.*

**Comments from Reviewer #1: Statistical review**

**This paper reports a retrospective analysis using propensity score weighting to investigate whether early methadone dose titration can improve outcomes compared to stable doses. Overall the statistical methods used are appropriate and the study is well reported. I had some comments on the paper, which are provided below:**

We thank the reviewer for their supportive comments and critical review of our manuscript.

1. **Abstract: I would recommend that the start of follow-up is defined in the abstract.**

Response: We thank the reviewer for highlighting the need for further clarity. We have included this information in the revised abstract.

Revised Text (Abstract):

*Dose titration between treatment days 4 to 6, with the index date defined as the first date of dose increase. For unexposed individuals, the index date was randomly assigned and followed the same distribution as those exposed.*

*Individuals were followed for up to 181 days following index date for our primary outcomes of methadone discontinuation and opioid toxicity, using an intention-to-treat approach.*

2. **Outcomes and follow-up: can the authors comment on the reliability of the outcome collection methods (especially the non-fatal ones).**

Response: Our primary outcome was non-fatal or fatal opioid toxicity, defined as an inpatient hospitalization or emergency department visit for opioid toxicity using International Classification of Diseases, 10th revision (ICD-10) diagnosis codes (T40.0–T40.4, T40.6) or opioid-related death using coronial records. For non-fatal toxicities, our study focused on hospital-treated events due to the lack of available data on toxicities treated in the community. However, it is possible that some non-fatal hospital-treated events were missed, particularly in cases where an opioid was not identified as contributing to the toxicity in the patient’s medical record. Given that the classification of a non-fatal opioid toxicity relies on patient self-reporting, toxicology testing or both, there is a potential for the underestimation of non-fatal opioid toxicities or potential misclassification of non-fatal opioid toxicities that were attributed to methadone versus non-methadone opioids. This is not a concern for our data on fatal opioid toxicities, as these data were obtained from the Chief Coroner of Ontario, which is based on post-mortem toxicology results. In Ontario, all sudden or unexpected deaths are investigated by a coroner, who is a trained physician, and as such this dataset is considered to be highly valid and robust. We have now added a limitation surrounding the potential under-reporting of non-fatal toxicities.

Revised Text (Discussion):

*Second, non-fatal opioid toxicities treated in the community without hospital transfer were not captured. Additionally, cases in which an opioid was not identified as the cause of toxicity in the patient’s medical report upon ED or hospital admission may have resulted in missed non-fatal toxicity events. Therefore, our findings may underestimate the overall incidence of non-fatal opioid toxicity in this population. Third, given defining the cause of a non-fatal toxicity relied on patient’s self-reporting, toxicology testing or both, there is potential for misclassification of non-fatal opioid toxicities that were attributed to methadone versus non-methadone opioids.*

3. **Statistical analysis: I assume all the variables used in the PS model pre-date the exposure time? Some sounds ambiguous on that (e.g. number of missed methadone doses, hospital visits etc). Later on the results, e.g. table 1, do clarify this is done correctly. I would recommend the text here is updated.**

Response: We appreciate the reviewer’s request for improved clarity regarding propensity score model covariates. We have since revised our definition of covariates in the main text. Due to the large number covariates included in our propensity score model we are unable to include all covariate definitions in the main text. We have provided all technical covariate definitions, including medical billing codes in S4 Table. If the reviewer and editor find it necessary to include these definitions in the main text, we are happy to make these changes.

Revised Text (methods, statistical analysis):

*We fit a propensity-score model to estimate the probability of dose titration, conditional on baseline demographic (age, sex, income quintile, housing status, urban residence, Northern Ontario residence), clinical (comorbidities, healthcare encounters for alcohol-benzodiazepine-, stimulant- and opioid-related toxicities, prior medication use), healthcare service use (non-OUD related outpatient visits, emergency department visits, hospital visits, attachment to primary care), methadone adherence (methadone dispense on the day prior to index, number of missed methadone doses between date of initiation and index, dose dispensed on initiation date, OUD-related outpatient visits between date of initiation and index) and prescriber related variables (physician specialty, prescriber OAT client volume, years in clinical practice) defined on or prior to index date (see S4 Table for covariate definitions).*

4. **Statistical analysis: I noted that it was mentioned missing data was grouped separately but I wasn't entirely sure what this meant. Can more be given on the missing data rates for variables and how these were dealt with in the PS model?**

Response: We appreciate the reviewer’s request for further clarity. We came across missing data for the following variables: income quintile (unexposed: 93 [1.7%] vs. exposed: 104 [1.2%]), location of urban residence (unexposed: 82 [1.5%] vs. 86 [1.0%]), and years in clinic practice (unexposed: 41 [0.7%] vs. exposed: 65 [0.8%]). These individuals were excluded from our analysis due to our inability to calculate a propensity score for people who missing data. Inaddition, we have revised the manuscript to clarify exclusion of observations due to propensity score trimming.

These details have been included in the revised text below. Lastly, we revised figure 1 to include missing data as an exclusion criteria.

Revised Text (Methods):

*Individuals with missing values for any covariates were excluded from our analysis.*

Revised Text (Results):

*After application of our exclusion criteria, we included 13,836 individuals in our study cohort (Figure 1). In the primary analysis, following propensity score trimming, the cohort comprised 13,560 individuals, of whom 8,322 had their methadone dose titrated early (i.e., between treatment days four and six; Table 1).*

*In the stratified analysis, after propensity score trimming, 5,123 individuals received a dose titration of <15mg (median, interquartile range dose titration [IQR]: 10mg, 10-10; S6 Table), while 3,173 were titrated by ≥15mg (median, IQR dose titration: 15mg, 15-15; S7 Table) on the index date.*

5. **Statistical analysis: "A 2-sided type I error rate of 0.05 was used for all comparisons" - since p-values are not reported, I am not sure this statement is needed? It could be replaced by a statement about 95% confidence intervals being used.**

Response: Thank you raising this important point. We have since revised the methods.

Revised Text (Methods):

*We used 95% confidence intervals (CI) to determine statistical significance, with estimates that crossed 1 considered statistically insignificant.*

6. **Results "After propensity-score weighting, all baseline characteristics were balanced between exposure groups (eFigure 1)." - table 1 also shows the SMDs so I would mention here.**

Response: Thank you highlighting the need for more information. We have since revised the text.

Revised text:

*After propensity-score weighting, all baseline characteristics were balanced between exposure groups with standardized differences of < 0.1.*

7. **Results: it would be useful to visualise the propensity score distribution (or weights) by exposed/non-exposed group to demonstrate that there was overlaps, meaning the positivity assumption was met.**

Response: We agree with the reviewer that inclusion of a propensity score distribution diagram will aid readers and demonstrate that our propensity score analysis met all required assumptions. We have since included this information in the text.

Revised Text:

*After propensity-score weighting, all baseline characteristics were balanced between exposure groups with standardized differences of < 0.1 (S1 Fig) and good propensity score distributional overlap between those exposed and unexposed was observed (S2 Fig).*

**Comments from Reviewer #2: Review of article: Association Between Early Methadone Dose Titration and Treatment Discontinuation  and Opioid Toxicity**

**General comments - 
This study examines an important area of methadone treatment that has had little exploration in the past - in spite of methadone being the most effective treatment for OUD for over 50 years, it remains unclear as to what is the most effective way to initiate and titrate the dose.  While lower doses may decrease the potential risk of methadone related toxicity and overdose, they may lead to continued use of illicit opioids and early treatment discontinuation.  This study is a good study that relates the dose titration early in methadone treatment episodes to outcomes - both retention in treatment and subsequent opioid toxicity, both from methadone and other opioids.  This information will be of critical importance to patients and medical providers when they start on methadone.  Overall, the study is well done, with a large database composed of many clinical and demographic measures.  I would strongly support publication.  Below are a few minor comments that might clarify some
questions.**

We thank the reviewer for their supportive comments and critical review of our manuscript.

1. **The paper briefly describes the protocols used in Canada and the US for initial methadone dose determinations in the discussion section.  Most US readers will not be familiar with the Canadian system. I would suggest providing a more complete description in the introduction - this will help readers understand why you chose days 4-6 as the exposure window.  US readers may not know that a physician visit is required for a dose increase and wonder why there aren't more dose increases in the first week.  They may also not be familiar with how dosing is done and how take-home doses are determined.**

Response: We thank the reviewer for raising the need for a brief discussion of the difference between methadone delivery in the US and Canada. We have revised the manuscript accordingly.

Revised Text (Introduction):

*In recent years, several US opioid treatment programs have adopted more aggressive titration schedules, with methadone doses increased daily, reaching doses of 70 mg or higher within the first week of treatment. In Ontario, Canada, the 2021 guidance by Mentoring, Education, and Clinical Tools for Addition - Partners in Health Integration (META-PHI) recommended initiating methadone at 30 mg rather than 20-30 mg as recommended under the former provincial guidelines, with dose increases of 15mg every 3-5 days until a dose of 75–80 mg per day is reached for people who use fentanyl. Although both US and Canada face a highly potent and volatile unregulated drug supply, their methadone delivery model differs. Specifically, in the US, methadone is exclusively prescribed and dispensed through federally certified opioid treatment programs, whereas in Canada, methadone is provincially regulated, commonly prescribed by outpatient clinicians (e.g., family physicians or nurse practitioners with an addiction medicine focused practice) and dispensed through community pharmacies. Because methadone prescribing and dispensing typically occur at separate locations in Canada, longstanding guidance recommends that prescribers conduct a follow-up assessment before increasing the dose to ensure lack of adverse effects (e.g., sedation).*

2. **The initial methadone dose on day 1 is given as a median - an average with calculation of standard difference would be more clarifying.**

Response: We appreciate the reviewers request for mean values and have included this information in Table 1.

3. **In figure 1, there were 93,357 "methadone use periods" - I assume this means this is the number of individuals who received any methadone in the time interval?  Although some patients might be listed more than once due to multiple treatment episodes?  Patients who were already on methadone (non-incident) were excluded.  Why was a methadone dose >60 excluded - is this any dose throughout the period above 60 mg, or just during initiation?  While this is mentioned in the text, it would help to put it on the figure as well.**

Response: We appreciate the reviewers request for further clarity. The reviewer is correct in their understanding that in creation of our cohort, we started with all methadone use periods identified within our accrual period of January 1, 2017, to June 30, 2023, and then excluded those not defined as incident use. Our definition of incidence use required no recent dispensing record for OAT or immediate release hydromorphone (e.g., safer opioid supply) within a predefined lookback period, which varied according to the drug formulation. In addition to this, we also excluded those dispensed methadone doses ≥60mg on the first day of treatment (i.e., initiation date), as methadone is typically not started at doses this high. Current guidelines in Canada recommend a starting dose of up 40mg on the first day of treatment depending on opioid tolerance to minimize risk of methadone toxicity. Therefore, it is likely that individuals dispensed doses ≥60mg on treatment day 1 are being misclassified as incident methadone recipients. For example, we are unable to identify methadone dispenses that occur out of province or within a correctional facility, once these individuals transition to receiving methadone from an Ontario community pharmacy, it may appear as if they have no prior history of methadone use. Lastly, we have also revised Figure 1, to clarify that we only excluded use periods dispensed methadone ≥60mg on initiation date.

4. **Under Exclusion Criteria, you indicate that for individuals with more than one exposure, one of these was chosen randomly.  Treatment retention declines with each subsequent treatment episode, so selecting from a group of episodes at random would have the effect of decreasing the average retention compared to the first recorded episode.  I would consider selecting the earliest treatment episode rather than a random selection.**

Response: We appreciate the reviewer suggestion and that retention on methadone may decline with each subsequent treatment episode as individuals may cycle on and off methadone over multiple years. Data access limits our look back to 2015, therefore, to prevent left censoring we did not select the first methadone treatment episode, as even though it may be the person’s first treatment episode over our study period, it is likely they received methadone prior to 2018. By selecting one use period per individual at random, we ensured that the distribution of prior episodes of methadone use is not skewed among our cohort.

5. **In Exclusion Criteria, you indicate that a methadone dose >60 mg was excluded (I assume for day 1, but it is not completely clear in the text), but don't indicate why this number was selected - the recommendation is for no more than 30 mg.  Under what circumstances in Canada is the initial dose more than 30 mg, or more than 60 mg?**

Response: Please see the response to comment 3.

6. **An indication of how take-home doses are determined in Canada is necessary to understand why this criterion was used.  The exclusion was applied at initiation - does this mean only the initial dose?  Over what period of time would a take-home dose be an exclusion?  Does Canada allow take-home doses on Sundays and holidays (as in the US)?**

Response: We thank the reviewer for raising this question. In Canada, take home doses are generally not granted until the individual has consistently received daily dispensed methadone for at least 14 days to 1 month. Given community-initiated methadone may be dispensed from any community pharmacy, provision of take-home doses on Sundays is not necessary. Take-home doses may be provided on statutory holidays when all pharmacies are closed (e.g., Christmas Day, Easter Monday) however, we did not account for this in our analysis. Similar to our rationale for excluding individuals dispensed doses ≥60mg on initiation, we chose to exclude those dispensed a take-home dose on initiation and on index date, as this may indicate that the individual is not an incident methadone recipient.

7. **Is there a good reason to exclude those <18 or >65 years old?  This criterion is not indicated in Figure 1.  How many were excluded on this basis?**

Response: We thank the reviewer for raising this question. The META-PHI prescribing guidance is generally not applicable to pediatric patients (<18 years) or older adults (>65 years). Specific dose titration recommendations for these populations are currently unavailable. Therefore, we restricted our analysis to the target population for the META-PHI methadone guidance. Lastly, only 382 incident methadone use periods were excluded due to age restrictions, as the majority of methadone use occurs among individuals aged 18 to 64. This information has been included to the revised flow chart.

8. **You define a methadone discontinuation as missing 6 consecutive days of dosing.  Why was this number chosen?  Your definition of prior exposure used a 30-day window.  Most OTPs in the US discharge patients after a 30-day dosing gap.  US OTPs usually institute a dose reduction for a few days for a treatment gap of 6 days but would not consider this to be a readmission.**

Response: We thank the reviewer for raising this question. The 30-day window was used to define incident methadone use to ensure capture of incident use periods that are all equally as likely to receive an early dose titration. According to Ontario-based META-PHI guidelines those with recent history of methadone use are recommended to have their doses titrated more rapidly than those without recent use. Based on clinical expertise we chose to define those with any history of methadone in the 30 days prior as recent methadone exposure. Alternatively, when defining methadone discontinuation, we used a six-day gap in treatment, as this duration reflects the point at which re-titration from the starting dose would be required. In Ontario, Canada, where the study was conducted, missing six consecutive days of methadone necessitates restarting treatment at the initial dose. We have revised the text to reflect this distinction.

Revised Text (Methods, Outcomes and Follow-up):

*We defined methadone discontinuation as a gap in treatment of six or more days which would require re-titration of methadone from its starting dose.*

9. **In the statistical analysis, the location of residence was used in the propensity adjustment, but it is not defined further in the text.  Table 1 appears to indicate that this means either residence in northern Ontario or not in addition to rural vs. urban.  What does it mean to be a resident of northern Ontario and why is it included?**

Response: We appreciate the reviewers request for further clarity regarding covariates included in the propensity score model. Covariates related to location of residence included 1) residence in rural vs. urban areas and 2) residence in Northern Ontario. We have now included this information in the main text. All covariates included in our propensity score model are also defined in S4 Table.

We included Northern Ontario residence in our propensity score model due to differences in the unregulated drug supply, OAT prescribing patterns and access to healthcare services in Northern versus Southern Ontario. Northern Ontario covers 90% of the provinces’ landmass and represents 6% of its population. The region is home to 856,193 people with more than half residing in its five largest centres. The remaining residents live in small and dispersed communities across the region. For this reason, sole inclusion of residence in urban areas was insufficient.

Revised Text (Methods, Statistical Analysis):

*We fit a propensity-score model to estimate the probability of dose titration, conditional on demographic (age, sex, income quintile, housing status, urban residence, Northern Ontario residence)*

10. **In the first sentence of Statistical Analysis, you state that "prior medication use" is a demographic variable - which medication does this refer to?**

Response: We appreciate the reviewer’s request for further clarity. Prior medication use refers to past history of controlled medications (benzodiazepines, stimulants, non-OAT opioids) in the 30 days prior to index date and direct acting antivirals or OAT (methadone, buprenorphine/naloxone, long-acting buprenorphine and SROM) in the year prior to index date. We point the reviewer S4 Table for a complete list and technical definitions of all covariates included in the propensity score model. If the reviewer and editor find it necessary to include this information in the main text, we are happy to make further revisions.

11. **The study uses the number of missed doses and a methadone dose dispensed prior to index in the propensity model.  In the US, the likelihood of a dose increase on any day is strongly dependent on whether there have been any recent missed doses.  A patient who misses a dose is unlikely to get dose increase on the following day.  Patients who miss dosing during the first few days may differ from those who dose every day in important ways that are not completely addressed in the propensity adjustment.  Would it be more accurate to simply exclude patients who missed doses prior to the index?**

Response: We agree with the reviewer, history of missed doses strongly influences the likelihood of a dose increase. For this reason we included a number of different methadone adherence covariates in our propensity score model to ensure adequate balance in the propensity to receive an early dose increase between exposure groups, such as receipt of methadone the day prior to index date, number of missed methadone doses between date of methadone initiation and index, methadone dose on initiation and outpatient physician visit after initiation and on/prior to index date. Additionally, we excluded individuals with >2 missed doses prior to assigned index date, due to low likelihood for receiving an early dose increase. Despite our best efforts, we agree with the reviewer that propensity score adjustment may not resolve all differences between those exposed and unexposed. As the reviewer has suggested we conducted a sensitivity among individuals with no missed doses prior to index date. Results from our sensitivity analysis were consistent with our main findings and are outlined in S9 Table and page 13 in the main text.

12. **In the stratified analysis, for the per-protocol analysis, there was an elevated risk of opioid toxicity between days 91 - 180 for those titrated by 15 or more mg (only related to non-methadone opioids).  This is not mentioned in the discussion - what would be a possible reason for this?**

Response: We are unable to determine with certainty what may be driving this finding, as several factors may contribute. The interval between days 91–180 is relatively distant from the index date, and many clinical and behavioral changes may occur by this time. This estimate was calculated among those still in treatment but given that the median duration of treatment was approximately three months, the sample size at this later interval is likely smaller. This is also reflected by the wider confidence interval compared with other point estimates (see S8 Table). Furthermore, individuals who were titrated by ≥15 mg and remained on treatment beyond 90 days may differ systematically from those censored earlier in ways we were unable to measure (e.g., OUD severity, continued use of the unregulated drug supply), which may confound our findings.

We did not discuss this finding in detail as it represents a single point estimate from a stratified analysis and was not a main focus of the study. However, if the editors feel that elaborating on this would strengthen the manuscript, we would be happy to include a brief discussion, recognizing that any interpretation would remain hypothesis-generating.

13. **Under limitations, since this is an observational study, it is not possible to prove a causal connection between a dose titration and retention, only a statistical association after adjustment for all observed parameters.  This is an important limitation of all non-randomized studies, and I think should be included in every case.**

Response: We agree with the reviewer and have added this as a limitation.

Revised Text (Discussion):

*Lastly, as with all observational studies due to the potential for residual confounding, we cannot be certain of a causal association between exposure and study outcomes.*

**Comments from Reviewer #3:**

**This paper describes a retrospective cohort study of methadone-treated patients, investigating the effects of early titration of the methadone dose. The research question is important, considering the on-going changes in patterns of opioid use, and the need for more data on different approaches to opioid agonist therapy. The manuscript is clearly written and easy to follow, and the basic methodology is sound (in the absence of experimental data). However, I have a couple of comments and questions:**

We thank the reviewer for their supportive comments and critical review of our manuscript.

1. **The authors mention in the introduction that some treatment providers use a very fast induction schedule, sometimes with daily methadone dose titration. Nevertheless, the authors have chosen to define "early dose titration" as occurring from day four to six. What about the patients who may have received an even earlier dose titration, before day four?**

Response: We thank the reviewer for their question. Because our data are Ontario-specific, we defined early dose titration as occurring between days four and six, based on the titration schedule outlined in the new methadone prescribing guidance released in Ontario, Canada. In addition, provision of dose titration prior to treatment day 4 was uncommon in our cohort, resulting in exclusion of 2,815 (7.2%) incident methadone use periods (N=39,356). Case reports outlining protocols and outcomes associated with daily dose titration were from opioid treatment programs in the US, which offer a different treatment model in comparison to Canada, where methadone is primarily dispensed from community pharmacies. I have revised the introduction to provide further details regarding the nuances between US and Canadian methadone regulations. Please refer to editor’s comment #1 and reviewer #2 comment #1 for revised text.

2. **I would be interested in knowing a bit more about the treatment of the unexposed patients, i.e. those that did not get any dose titration during the first six days. If I interpret eTable 7 correctly, 54.3% of them got a dose increase within 14 days of the index date (which corresponds to days 18-20 from treatment initiation). Isn't this a bit surprising? Where I work, we rarely wait more than a week before beginning the titration, and in this cohort almost half of the patients had to wait more than 2.5 weeks? Perhaps the requirement to meet a physician in order to change the dose, that is mentioned in the Discussion, might result in a sub-optimal dosing schedule even for some patients with "normal" titration rates? Maybe a comparison between early (<7 days), standard, and late (>14 days) titration starts would be of interest?**

Response: We thank the reviewer for raising the need for further clarity. The reviewer is correct in their interpretation. We determined provision of a subsequent dose titration within 14 days of index date (i.e., up to treatment days 18-20). In this exploratory analysis, we found that 54.4% of those unexposed were provided a dose titration with a median time to dose increase of 4 days (IQR: 3-6) versus 76.8% of those exposed (S10 Table). We agree with the reviewer that waiting nearly two weeks for a dose increase is not an optimal dosing strategy, which is why our study likely found an increased hazard of methadone discontinuation observed among those unexposed. Specifically, within the first 7 days following index date, the hazard of methadone discontinuation was 0.55 (95% CI: 0.51, 0.60) among those exposed versus unexposed. Lastly, as this was an ad-hoc analysis, we cannot conduct additional analysis comparing early, standard and late titration without reframing our study cohort and index date.

Overall, we agree with the reviewer that waiting nearly two weeks for an initial methadone dose increase represents a highly suboptimal dosing schedule. However, physicians may sometimes be limited in their ability to titrate the dose if patients miss doses, given the risk of methadone toxicity. In such cases, clinicians may choose to maintain the patient on the initial dose as a harm reduction measure. We have included the need for future qualitative studies to identify modifiable barriers which may prevent timely provision of dose titration.

Revised Text (Discussion):

*Future qualitative research should also explore barriers to dose titration to identify modifiable factors that may be addressed to improve titration rates and support safer, more effective methadone initiation.*

3. **The authors mention a new guideline that was issued in 2021, recommending an early dose increase for people using fentanyl. However, the study period was from 2017 to 2022, which makes me wonder if the apparent effects of the exposure could be confounded by time, if most early titration cases occurred toward the end of the study period? I am not very familiar with the specific situation in Canada, but I imagine that changes in the drug market, as well as factors related to the covid-19-pandemic etc., could have resulted in different outcomes for this population over different parts of the study period.**

Response: We appreciate the reviewer’s concern regarding time-related bias. We confirm that the year of the index date was included as a covariate in our propensity score model (see S4 Table a full list of covariates), and have revised table 1, S6 Table and S7 Table to include this information. Based on these results, the identification of incident methadone use periods that received early dose titration was well balanced across all study years, and any imbalances between those exposed versus unexposed were resolved after implementation of inverse propensity score weighting.

**In summary, this is an interesting study of an important clinical question. If the authors could clarify the questions above, I believe the manuscript could be suitable for publication.**

Response: Thank you for supporting our manuscript.

**Technical Comments**

1. **Please send a completed 'Competing Interests' statement, including any COIs declared by your co-authors. If you have no competing interests to declare, please state "The authors have declared that no competing interests exist". Otherwise please declare all competing interests beginning with the statement "I have read the journal's policy and the authors of this manuscript have the following competing interests:"**

Response: Thank you for bringing this to our attention. We have now included a complete competing interest statement.

2. **Please amend your detailed Financial Disclosure statement. This is published with the article. It must therefore be completed in full sentences and contain the exact wording you wish to be published.**

Response: Thank you for bringing this to our attention. We have since amended our financial disclosure statement.

3. **State what role the funders took in the study. If the funders had no role in your study, please state: “The funders had no role in study design, data collection and analysis, decision to publish, or preparation of the manuscript.”**

Response: Thank you for bringing this to our attention. We have included the above statement in our amended financial disclosure.

4. **If any authors received a salary from any of your funders, please state which authors and which funders.**

Response: No authors received a salary from any of the funders.

5. **Please provide separate figure files in .tif or .eps format.**

Response: Completed.

6. **We have noticed that you have uploaded Supporting Information files, but you have not included a list of legends. Please add a full list of legends for your Supporting Information files after the references list.**

Response: Thank you for bringing this to our attention. We have included a list of legends in our revised manuscript.

**General editorial requests:**
(Note: not all will apply to your paper, but please check each item carefully)

1. We ask every co-author listed on the manuscript to fill in a contributing author statement, making sure to declare all competing interests. If any of the co-authors have not filled in the statement, we will remind them to do so when the paper is revised. If all statements are not completed in a timely fashion this could hold up the re-review process. If new competing interests are declared later in the revision process, this may also hold up the submission. Should there be a problem getting one of your co-authors to fill in a statement we will be in contact. Please do not add or remove authors without first discussing this with the handling editor.

Response: Thank you for alerting us of this.

2. Please upload any figures associated with your paper as individual TIF or EPS files with 300dpi resolution at resubmission.

Response: Complete.

3. Please ensure that the paper adheres to the PLOS Data Availability Policy, which requires that all data underlying the study's findings be provided in a repository or as Supporting Information. For data residing with a third party, authors are required to provide instructions with contact information (web or email address) for obtaining the data. Please note that a study author cannot be the contact person for the data. PLOS journals do not allow statements supported by "data not shown" or "unpublished results." For such statements, authors must provide supporting data or cite public sources that include it.

Response: A complete data availability statement has been provided.

4. We expect all researchers with submissions to PLOS in which author-generated code underpins the findings in the manuscript to make all author-generated code available without restrictions upon publication of the work. In cases where code is central to the manuscript, we may require the code to be made available as a condition of publication. Authors are responsible for ensuring that the code is reusable and well documented. Please make any custom code available, either as part of your data deposition or as a supplementary file. Please add a sentence to your data availability statement regarding any code used in the study, e.g. "The code used in the analysis is available from Github [URL] and archived in Zenodo [DOI link]"

Response: Not applicable.

5. The funding statement should include: specific grant numbers, initials of authors who received each award, URLs to sponsors’ websites. Also, please state whether any sponsors or funders (other than the named authors) played any role in study design, data collection and analysis, the decision to publish, or preparation of the manuscript. If they had no role in the research, include this sentence: “The funders had no role in study design, data collection and analysis, decision to publish, or preparation of the manuscript.”

Response: This information is provided in the financial disclosures statement.

**FORMATTING - GENERAL**

6. Abstract: Please structure your abstract using the PLOS Medicine headings (Background, Methods and Findings, Conclusions). Please combine the Methods and Findings sections into one section.

Response: Complete.

7. At this stage, we ask that you include a short, non-technical Author Summary of your research to make findings accessible to a wide audience that includes both scientists and non-scientists. The Author Summary should immediately follow the Abstract in your revised manuscript. This text is subject to editorial change and should be distinct from the scientific abstract. Ideally each sub-heading should contain 2-3 single sentence, concise bullet points containing the most salient points from your study. In the final bullet point of 'What Do These Findings Mean?', please include the main limitations of the study in non-technical language.

Response: Complete.

8. Please express the main results with 95% CIs as well as p values. When reporting p values please report as p<0.001 and where higher as the exact p value p=0.002, for example. Throughout, suggest reporting statistical information as follows to improve clarity for the reader "22% (95% CI [13%,28%]; p</=)". Please be sure to define all numerical values at first use.

Response: We reported confidence intervals throughout our manuscript. If the editors prefers the inclusion of p-values, we are happy to include them.

9. Please include page numbers and line numbers in the manuscript file. Use continuous line numbers (do not restart the numbering on each page).

Response: Completed.

10. Please cite the reference numbers in square brackets. Citations should precede punctuation.

Response: Unfortunately, the citation software we used does not provide an option to format reference numbers in square brackets. Would it be acceptable to submit the manuscript with citations in parentheses instead? If not, my team and I can troubleshoot the formatting issue or manually replace the parentheses with brackets prior to submission.

**FIGURES AND TABLES**

11. Please provide titles and legends for all figures and tables (including those in Supporting Information files).

Response: Completed.

12. Please define all abbreviations used in each figure/table (including those in Supporting Information files).

Response: Completed.

13. Please consider avoiding the use of red and green in order to make your figure more accessible to those with color blindness.

Response: We confirm no use of red and green in figures.

**SUPPLEMENTARY MATERIAL**

14. Please note that supplementary material will be posted as supplied by the authors. Therefore, please amend it according to the relevant comments outlined here.

Response: Thank you for raising this important point. We confirm all supplementary material was been amended according to relevant comments.

15. Please cite your Supporting Information as outlined here: [https://can01.safelinks.protection.outlook.com/?url=https%3A%2F%2Fjournals.plos.org%2Fplosmedicine%2Fs%2Fsupporting-information&data=05%7C02%7Cria.garg%40mail.utoronto.ca%7Ca6307017398f4cdf91ae08ddff69f328%7C78aac2262f034b4d9037b46d56c55210%7C0%7C0%7C638947552710495903%7CUnknown%7CTWFpbGZsb3d8eyJFbXB0eU1hcGkiOnRydWUsIlYiOiIwLjAuMDAwMCIsIlAiOiJXaW4zMiIsIkFOIjoiTWFpbCIsIldUIjoyfQ%3D%3D%7C0%7C%7C%7C&sdata=37iSbMyGqICey8ALvwADPS%2BJ4X9BtYIAsH2h%2Fs8vwqA%3D&reserved=0](https://can01.safelinks.protection.outlook.com/?url=https%3A%2F%2Fjournals.plos.org%2Fplosmedicine%2Fs%2Fsupporting-information&data=05|02|ria.garg@mail.utoronto.ca|a6307017398f4cdf91ae08ddff69f328|78aac2262f034b4d9037b46d56c55210|0|0|638947552710495903|Unknown|TWFpbGZsb3d8eyJFbXB0eU1hcGkiOnRydWUsIlYiOiIwLjAuMDAwMCIsIlAiOiJXaW4zMiIsIkFOIjoiTWFpbCIsIldUIjoyfQ%3D%3D|0|||&sdata=37iSbMyGqICey8ALvwADPS%2BJ4X9BtYIAsH2h%2Fs8vwqA%3D&reserved=0)

Response: Thank you, we have cited supported information according to provided instructions.

**REFERENCES**

16. PLOS uses the numbered citation (citation-sequence) method and first six authors, et al.

Response: Unfortunately, the citation software we used does not provide an option to format reference numbers in square brackets. Would it be acceptable to submit the manuscript with citations in parentheses instead? If not, my team and I can troubleshoot the formatting issue or manually replace the parentheses with brackets prior to submission.

17. Please ensure that journal name abbreviations match those found in the National Center for Biotechnology Information (NCBI) databases ([https://can01.safelinks.protection.outlook.com/?url=http%3A%2F%2Fwww.ncbi.nlm.nih.gov%2Fnlmcatalog%2Fjournals&data=05%7C02%7Cria.garg%40mail.utoronto.ca%7Ca6307017398f4cdf91ae08ddff69f328%7C78aac2262f034b4d9037b46d56c55210%7C0%7C0%7C638947552710513822%7CUnknown%7CTWFpbGZsb3d8eyJFbXB0eU1hcGkiOnRydWUsIlYiOiIwLjAuMDAwMCIsIlAiOiJXaW4zMiIsIkFOIjoiTWFpbCIsIldUIjoyfQ%3D%3D%7C0%7C%7C%7C&sdata=szoRKFoVD96yA6cBSYbnH3XnCrv1q2yAUp2ueC7yC9U%3D&reserved=0](https://can01.safelinks.protection.outlook.com/?url=http%3A%2F%2Fwww.ncbi.nlm.nih.gov%2Fnlmcatalog%2Fjournals&data=05|02|ria.garg@mail.utoronto.ca|a6307017398f4cdf91ae08ddff69f328|78aac2262f034b4d9037b46d56c55210|0|0|638947552710513822|Unknown|TWFpbGZsb3d8eyJFbXB0eU1hcGkiOnRydWUsIlYiOiIwLjAuMDAwMCIsIlAiOiJXaW4zMiIsIkFOIjoiTWFpbCIsIldUIjoyfQ%3D%3D|0|||&sdata=szoRKFoVD96yA6cBSYbnH3XnCrv1q2yAUp2ueC7yC9U%3D&reserved=0)), and are appropriately formatted and capitalised.

Response: We confirm citations have been provided according to provided instructions.

18. Where website addresses are cited, please include the complete URL and specify the date of access (e.g. [accessed: 12/06/2023]).

Response: We confirm citations have been provided according to provided instructions.

19. Please also see [https://can01.safelinks.protection.outlook.com/?url=https%3A%2F%2Fjournals.plos.org%2Fplosmedicine%2Fs%2Fsubmission-guidelines%23loc-references&data=05%7C02%7Cria.garg%40mail.utoronto.ca%7Ca6307017398f4cdf91ae08ddff69f328%7C78aac2262f034b4d9037b46d56c55210%7C0%7C0%7C638947552710531011%7CUnknown%7CTWFpbGZsb3d8eyJFbXB0eU1hcGkiOnRydWUsIlYiOiIwLjAuMDAwMCIsIlAiOiJXaW4zMiIsIkFOIjoiTWFpbCIsIldUIjoyfQ%3D%3D%7C0%7C%7C%7C&sdata=TYjLD6I%2FITMu9CSBqc8UF82lTLoae%2FRo1j7zpEycN6A%3D&reserved=0](https://can01.safelinks.protection.outlook.com/?url=https%3A%2F%2Fjournals.plos.org%2Fplosmedicine%2Fs%2Fsubmission-guidelines%23loc-references&data=05|02|ria.garg@mail.utoronto.ca|a6307017398f4cdf91ae08ddff69f328|78aac2262f034b4d9037b46d56c55210|0|0|638947552710531011|Unknown|TWFpbGZsb3d8eyJFbXB0eU1hcGkiOnRydWUsIlYiOiIwLjAuMDAwMCIsIlAiOiJXaW4zMiIsIkFOIjoiTWFpbCIsIldUIjoyfQ%3D%3D|0|||&sdata=TYjLD6I%2FITMu9CSBqc8UF82lTLoae%2FRo1j7zpEycN6A%3D&reserved=0) for further details on reference formatting.

Response: Thank you for this response.

**STUDY TYPE-SPECIFIC REQUESTS**

20. Abstract: Please include the study design, population and setting, number of participants, years during which the study took place (enrollment and follow up), length of follow up, and main outcome measures.

Response: We confirm all require information is included in the abstract.

21. Please ensure that the study is reported according to the STROBE (or appropriate STOBE extension) guideline (available from: [https://can01.safelinks.protection.outlook.com/?url=https%3A%2F%2Fwww.equator-network.org%2Freporting-guidelines%2Fstrobe&data=05%7C02%7Cria.garg%40mail.utoronto.ca%7Ca6307017398f4cdf91ae08ddff69f328%7C78aac2262f034b4d9037b46d56c55210%7C0%7C0%7C638947552710547695%7CUnknown%7CTWFpbGZsb3d8eyJFbXB0eU1hcGkiOnRydWUsIlYiOiIwLjAuMDAwMCIsIlAiOiJXaW4zMiIsIkFOIjoiTWFpbCIsIldUIjoyfQ%3D%3D%7C0%7C%7C%7C&sdata=dCkpvOBqZw0MGS28Tmn1I%2BZF5bnKc85H5XDJo95sd8E%3D&reserved=0)](https://can01.safelinks.protection.outlook.com/?url=https%3A%2F%2Fwww.equator-network.org%2Freporting-guidelines%2Fstrobe&data=05|02|ria.garg@mail.utoronto.ca|a6307017398f4cdf91ae08ddff69f328|78aac2262f034b4d9037b46d56c55210|0|0|638947552710547695|Unknown|TWFpbGZsb3d8eyJFbXB0eU1hcGkiOnRydWUsIlYiOiIwLjAuMDAwMCIsIlAiOiJXaW4zMiIsIkFOIjoiTWFpbCIsIldUIjoyfQ%3D%3D|0|||&sdata=dCkpvOBqZw0MGS28Tmn1I%2BZF5bnKc85H5XDJo95sd8E%3D&reserved=0)) and include the completed STROBE (or STROBE extension) checklist as Supporting Information. Please add the following statement, or similar, to the Methods: "This study is reported as per the Strengthening the Reporting of Observational Studies in Epidemiology (STROBE) guideline (S1 Checklist)." When completing the checklist, please use section and paragraph numbers, rather than page numbers.

Response: We confirm inclusion of the completed STROBE checklist and statement.

22. Please check whether the RECORD guideline (available from [https://can01.safelinks.protection.outlook.com/?url=https%3A%2F%2Fwww.record-statement.org%2F&data=05%7C02%7Cria.garg%40mail.utoronto.ca%7Ca6307017398f4cdf91ae08ddff69f328%7C78aac2262f034b4d9037b46d56c55210%7C0%7C0%7C638947552710564785%7CUnknown%7CTWFpbGZsb3d8eyJFbXB0eU1hcGkiOnRydWUsIlYiOiIwLjAuMDAwMCIsIlAiOiJXaW4zMiIsIkFOIjoiTWFpbCIsIldUIjoyfQ%3D%3D%7C0%7C%7C%7C&sdata=VnaWLvlMuUdHYhsytKUN8yjDxOWTsPf0BtdZHXGbonE%3D&reserved=0)](https://can01.safelinks.protection.outlook.com/?url=https%3A%2F%2Fwww.record-statement.org%2F&data=05|02|ria.garg@mail.utoronto.ca|a6307017398f4cdf91ae08ddff69f328|78aac2262f034b4d9037b46d56c55210|0|0|638947552710564785|Unknown|TWFpbGZsb3d8eyJFbXB0eU1hcGkiOnRydWUsIlYiOiIwLjAuMDAwMCIsIlAiOiJXaW4zMiIsIkFOIjoiTWFpbCIsIldUIjoyfQ%3D%3D|0|||&sdata=VnaWLvlMuUdHYhsytKUN8yjDxOWTsPf0BtdZHXGbonE%3D&reserved=0)) and include the completed checklist as Supporting Information might be more suitable for your study type.

Response: Not applicable.

23. For all observational studies, in the manuscript text, please indicate: (1) the specific hypotheses you intended to test, (2) the analytical methods by which you planned to test them, (3) the analyses you actually performed, and (4) when reported analyses differ from those that were planned, transparent explanations for differences that affect the reliability of the study's results. If a reported analysis was performed based on an interesting but unanticipated pattern in the data, please be clear that the analysis was data driven.

Response: We confirm all required information is provided in the manuscript.

24. Please state in the Methods section whether the study had a prospective protocol or analysis plan. If a prospective analysis plan (from your funding proposal, IRB or other ethics committee submission, study protocol, or other planning document written before analyzing the data) was used in designing the study, please include the relevant document(s) with your revised manuscript as a Supporting Information file to be published alongside your study and cite it in the Methods section. A legend for this file should be included at the end of your manuscript. If no such document exists, please make sure that the Methods section transparently describes when analyses were planned, and when/why any data-driven changes to analyses took place. Changes in the analysis, including those made in response to peer review comments, should be identified as such in the Methods section of the paper, with rationale.

Response: Thank you for raising this important point. We confirm that we have cited our pre-registered analysis in the methods section.
