# Supplementary material for: Association between early methadone dose titration and treatment discontinuation and opioid toxicity: A retrospective cohort study
Source: PLoS Med. 2026 Apr 9;23(4):e1004748. doi: 10.1371/journal.pmed.1004748 (PMC13065010; doi:10.1371/journal.pmed.1004748)
Supplement: Attachment — Submitted filename: Reviewer response_2025Dec30.docx [file pmed.1004748.s017.docx]

**Comments from the academic editor:**

1. **The authors made a conclusion that high dose methadone (>60mg) should be excluded as a starting dose and yet it is high dose inductions that is the focus of the study - I wonder whether the authors might need to do some additional analysis - albeit post hoc - to consider a) whether inclusion of the high doses they have excluded changes the observed findings, b) whether the high dose initiations look different to the lower dose ones in terms of characteristics of the people or treatment episodes (i.e. any basis for their assumption that these are episodes continued from out of jurisdiction or incarceration) and c) whether there is a time pattern observed whereby proportionally more of these occur in later years of the study - since dose is the object of focus of the study and this is a significant limitation in capture of the dataset. I think it would be helpful to do this additional investigation.**

**Response:** We thank the editor for this thoughtful comment. We agree that understanding the characteristics of individuals receiving a higher methadone dose initiation is important. However, we have several clinical and methodological reasons for maintaining the exclusion of individuals initiated at methadone doses ≥60mg. We have addressed our specific reasons below:

1. Clinical Rationale and Adherence to Guidelines: The primary objective of our study is to evaluate the safety and effectiveness of early dose titration within an outpatient setting, as outlined by current prescribing guidelines. Clinical guidance across Canada and the US recommends initiation of methadone at doses <60mg in an outpatient setting, even for people who use highly potent opioids such fentanyl. Specifically, due to methadone’s long half-live and incomplete opioid cross-tolerance, initiation at a dose of 60mg or higher for those who are methadone naïve would increase their risk of methadone toxicity and respiratory depression. Specifically, current guidance in BC and Ontario, Canada suggests a **maximum initiation dose** of **40mg**, while SAMHSA in the US recommends up to maximum of **50mg** within an outpatient setting. Therefore, doses exceeding 50mg are expected to be exceedingly rare in the community, meaning that our exclusion of those with a first observed dose of 60mg is unlikely to be excluding anyone truly initiating a new course of methadone treatment. Instead, these individuals are most likely prevalent users. Finally, even in the rare event that someone was started at a dose above 60mg, this would be considered a “high dose initiation” misaligned with standard clinical practice, which should not be conflated with "rapid dose increases," which occurs when doses are increased rapidly following initiation.

2. Risk of Misclassification: As described above, individuals with a first outpatient prescription with a dose over 60mg are most likely prevalent users misclassified as incident users due to limitations of our administrative databases. Specifically, our inability to capture methadone dispensing that occurs within federal or provincial correctional facilities or in other provincial jurisdictions. When an individual transitions from a correctional facility or moves to Ontario while already stabilized on a high dose of methadone, their first record in an Ontario-based community pharmacy database appears as a "new" start at a high dose. Therefore, we believe that the risk of misclassification bias by including people likely to be prevalent methadone recipients is high, leading to our decision to maintain this exclusion criterion.

3. Feasibility of Post Hoc Analysis: Finally, while we carefully considered conducting a post-hoc analysis to compare characteristics of people who initiated methadone at doses ≥60mg and <60mg, we believe it would not provide the clarity the reviewer seeks for the following reasons:

- Data Resolution: We do not have access to data which may indicate incarceration dates or whether they resided in another province prior to receiving methadone in Ontario, Canada.
- Resource Requirements: Because these individuals were excluded at the earliest stage of cohort creation to ensure the integrity of the "incident use" definition, re-extracting and cleaning data for this specific sub-group would require a significant investment of time and resources without a guarantee of meaningful findings given the rationale provided in #1 and #2 above.

Therefore, we have strengthened the limitations section in our revised manuscript to acknowledge that - while there is a small risk that this exclusion removes a small number of people truly initiating very high-dose methadone, this was a necessary step to minimize the misclassification bias from including prevalent methadone users, and to maintain consistency of our study with Canadian and U.S. methadone prescribing guidance within an outpatient setting. See revised text below.

Revised Text: *Sixth, we excluded treatment episodes initiated at doses ≥60mg to ensure our cohort reflected true incident use and was aligned with current methadone prescribing guidance. While this may have excluded a small number of people starting methadone at very high doses, Canadian guidelines generally recommend a maximum starting dose of 40mg in outpatient settings to mitigate the risk of methadone toxicity. Therefore, these cases most likely represent prevalent users whose prior dispensing history was not captured in our dataset (e.g., those transitioning from correctional facilities or moving to Ontario from other provinces).*

2. **Additionally, the justification for selecting a random treatment episode (rather than the first) does not seem entirely robust. Selecting a random event means that you will randomly usually select a subsequent episode not a first one - at least if you use the first observed episode, you're minimising the number of times where you are using a repeated treatment entry that the authors claim would be at play given the truncation of the observation period?

   Editorial note: We consulted the statistical editor regarding the second comment, and they agree with the academic editor's point. They agree that your justification for using random selection instead of an alternative method (e.g., the first eligible visit) is insufficient. They suggest conducting a sensitivity analysis using an alternative method, such as the first visit; we strongly encourage you to perform this analysis.**

**Response:** We thank the editor for this comment. We agree addition of a post-hoc analysis using the first methadone initiation captured within our accrual period will help strengthen our findings and have included this as an exploratory sensitivity analysis in our manuscript. The overall results were generally consistent with our primary analysis. See revised text and results of the additional exploratory analysis (S10 Table) below.

Revised Text (Methods): *Lastly, we reconstructed the study cohort to include each individual’s first continuous use period accrued during the exposure window and reanalyzed the primary study outcomes to test the robustness of our decision to randomly select one incident use period per person.*

Revised Text (Results): *Results from our sensitivity analyses were generally consistent with the main findings and were robust to changes in our definition of methadone discontinuation, cohort restrictions and selection of a unique continuous use period at random versus the first incident use period identified over the accrual period (S9 Table and S10 Table).*

**S10 Table. Association between early dose titration and study outcomes, exploratory analysis^a^**

| **Outcome** | **Rate^b^ per 100 person-years (95% CI)** | | **Hazard Ratio^b,c^ (95% CI)** |
| --- | --- | --- | --- |
|  | **Unexposed**  **(No dose increase)** | **Exposed**  **(Dose increase)** |  |
| **Methadone Discontinuation** | 289.59  (276.0, 303.9) | 207.0  (200.1, 214.2) | Interval 1^d^: 0.53 (0.48, 0.57) |
|  |  |  | Interval 2^e^: 0.80 (0.74, 0.87) |
|  |  |  | Interval 3^f^: 0.83 (0.76, 0.90) |
|  |  |  | Interval 4^g^: 0.87 (0.78, 0.96) |
| **Opioid toxicity** | | | |
| Intention to treat | 11.0 (9.6, 12.5) | 9.7 (8.7, 10.9) | 0.89 (0.75, 1.05) |
| While on treatment | 7.5 (6.0, 9.2) | 5.5 (4.7, 6.5) | 0.81 (0.62, 1.06) |
| Methadone toxicity  (while on treatment) | 2.4 (1.7, 3.5) | 2.0 (1.5, 2.7) | 0.92 (0.58, 1.45) |
| Non-methadone toxicity  (while on treatment) | 6.7 (5.2, 8.6) | 5.9 (5.0, 7.1) | 0.93 (0.69, 1.26) |

**Foot Notes:**

^a^Study cohort includes first incident use period identified per person

^b^stabilised inverse probability treatment weighting

^c^Reference group: Unexposed

^d^0 to 7 days of follow-up

^e^8 to 30 days of follow-up

^f^31 to 90 days of follow-up

^g^91 to 181 days of follow-up

CI, confidence interval

**Comments from the reviewers:** 

**Reviewer #1: Thank you to the authors for fully addressing my previous review comments. I have no further issues to raise.**

**Response:** We thank the reviewer for appraising our manuscript.

**Reviewer #2: Thank you for your detailed responses.  Thank you also for your contribution to this important area.  I have no more comments or revisions at this time.**

**Response:** We thank the reviewer for their kind words.

**Reviewer #3: The authors have made a thorough revision of the manuscript, with a number of changes that made it stronger and improved clarity. I agree with the other reviewer comments, that it is important to contextualize the findings, for readers like me that are less familiar with the specifics of the Canadian treatment system. I believe that this version of the manuscript makes this more clear, and I have no further comments to add at this point.**

**Response:** We thank the reviewer for appraising our manuscript.

**Requests from Editors:**

**GENERAL**

1. **Please confirm that your title complies with to PLOS Medicine's style. Your title must be nondeclarative and not a question. It should begin with main concept if possible. "Effect of" should be used only if causality can be inferred, i.e., for an RCT. Please place the study design ("A randomized controlled trial," "A retrospective study," "A modelling study," etc.) in the subtitle (ie, after a colon).**

**Response:** Thank you for bringing this to our attention. Please have revised the title and subtitle accordingly.

Revised Text:

*Title: Association Between Early Methadone Dose Titration and Treatment Discontinuation and Opioid Toxicity: A retrospective cohort study*

*Subtitle: Early methadone dose titration and discontinuation and opioid toxicity: A retrospective cohort study*

2. **Statistical reporting: Please revise throughout the manuscript, including tables and figures.**
   - **Please report statistical information as follows to improve clarity for the reader ""22% (95% CI [13,28]; p</=)"".**
   - **Please separate upper and lower bounds with commas instead of hyphens as the latter can be confused with reporting of negative values.**
   - **Please repeat statistical definitions (HR, CI etc.) for each set of parentheses.**

**Response:** We confirm that we have revised statistical reporting throught the manuscript and tables.

3. **Please ensure that all abbreviations are defined at first use throughout the text (including statistical abbreviations).**

**Response:** We confirm all abbreviations are defined at first use throughout the manuscript.

4. **Please ensure that tables and figures, including those in supplementary files, are appropriately referenced in the main text.**

**Response:** Thank you for bringing this to our attention. We confirm all tables, figures and supplementary files are appropriately referenced in the main text.

5. **Please review your text for claims of novelty or primacy (e.g. 'for the first time' or ‘novel’) and remove this language.**

**Response:** We confirm that no have not claimed novelty or primacy at any point in our manuscript.

6. **Please confirm that any use of statistical terms (such as trend or significant) are supported by the data, and if not please remove them. The term trend should be used only when the test for trend has been conducted.**

**Response:** We agree with above and have ensured all use of statistical terms are supported by data and statistical analysis results.

7. **Please provide titles and legends for all figures and tables (including those in Supporting Information files).**

**Response:** We confirm that we have provided titles and legends for all figures and tables.

8. **Please define all acronyms used in each figure or table in its corresponding legend.**

**Response:** We confirm that we have defined all acronyms used in each figure or table in its corresponding legend.

9. **Please confirm the use of patient-centered language. Please note that patient-centered language is constructed with the use of post-modified nouns putting the person first in the sentence structure.**

**Response:** We confirm use of patient-centered language.

10. **Your study is observational and therefore causality cannot be inferred. Please check your manuscript and confirm that you have not used language that implies causality.**

**Response:** We confirm that we did not use any language, which may imply causality.

11. **Please consider including an Acknowledgments section in your manuscript, acknowledging the study participants as well as the individuals who played a role in data collection or participant care or other involvement.**

**Response:** We confirm that we have acknowledged all people and/or organizations that support our study.

**ABSTRACT**

12. **Please confirm that your abstract complies with our requirements, including providing all the information relevant to this study type**

<https://can01.safelinks.protection.outlook.com/?url=https%3A%2F%2Fjournals.plos.org%2Fplosmedicine%2Fs%2Fsubmission-guidelines%23loc-abstract&data=05%7C02%7Cria.garg%40mail.utoronto.ca%7C0cfe141e8999421a910e08de3e5528d2%7C78aac2262f034b4d9037b46d56c55210%7C0%7C0%7C639016732647727459%7CUnknown%7CTWFpbGZsb3d8eyJFbXB0eU1hcGkiOnRydWUsIlYiOiIwLjAuMDAwMCIsIlAiOiJXaW4zMiIsIkFOIjoiTWFpbCIsIldUIjoyfQ%3D%3D%7C0%7C%7C%7C&sdata=zxzd2D89B1%2FIbtyVJqeayQMA16yBgJr35u8oE8WwcLY%3D&reserved=0>

**Response:** We confirm that we have included all required information in our abstract.

13. Please confirm that all numbers presented in the abstract are present and identical to numbers presented in the main manuscript text.

**Response:** We confirm that all numbers presented in the abstract are present and identical to numbers presented in the main manuscript text.

14. In the abstract, please include the important dependent variables that are adjusted for in the analyses.

**Response:** We confirm we have included important dependent variables that are adjusted for in the analyses. See revised text below.

Revised Text: *We fit a propensity-score model to estimate the probability of dose titration, conditional on baseline demographic and clinical (e.g., comorbidities, past medication use) variables. Propensity score weighted Cox proportional hazard models were then estimated to determine the association between early dose titration and study outcomes.*

15. In the last sentence of the Abstract Methods and Findings section, please describe the main limitation(s) of the study's methodology.

**Response:** Thank you for bringing this to our attention. We confirm we have now included the main study limitation to the abstract. See below for revised text.

Revised Text: *The main study limitation is the lack of information on drug use history and time-varying clinical factors that may influence both dose titration and subsequent outcomes, introducing the potential for residual confounding.*

AUTHOR SUMMARY

16. **“Among people who use a fentanyl-dominated unregulated drug supply, the provision of an early methadone dose titration supported methadone retention, which appears to have led to reduced opioid toxicity.” – please avoid claims of causality.**

**Response:** Thank you for bringing this to our attention. We have revised the sentence to remove any claims of causality. Please below revised text.

Revised Text: Logistical barriers that prevent people with high opioid tolerance from receiving timely dose titration need to be addressed given the association of early dose titration with improved methadone retention and lower rates of opioid toxicity.

17. **In the author summary, in the final bullet point of 'What Do These Findings Mean?', please include the main limitations of the study in non-technical language.**

**Response:** Thank you for bringing this to our attention. We have included main study limitations in the appropriate section of the manuscript. See revised text below.

Revised Text: *The study’s main limitation was lack of comprehensive data on clinical indicator of opioid use disorder severity such as their drug use history or the type of drugs used.*

**METHODS AND RESULTS**

18. **Thank you for providing your STROBE checklist. Please replace the page numbers with paragraph numbers per section (e.g. "Methods, paragraph 1"), since the page numbers of the final published paper may be different from the page numbers in the current manuscript.**

**Response:** Thank you for raising this important point. We have revised the STROBE checklist.

19. **Please confirm that you provided the unadjusted comparisons as well as the adjusted comparisons in all relevant Tables.**

**Response:** We have included unadjusted event rates and hazard ratios for our primary analysis in the appendix. See S5 Table for the unadjusted comparisons.

20. **Please confirm that you specified the variables controlled for in all relevant Tables.**

**Response:** We confirm that we have specified whether results were unadjusted or propensity score weighted in all relevant tables.

**DISCUSSION**

21. **Please remove the 'conclusions' subheading from the discussion. Please also remove any other subheadings from the discussion.**

**Response:** Thank you for bringing this to our attention. We have removed all subheadings from the discussion.

**General Editorial Requests**

22. **We ask every co-author listed on the manuscript to fill in a contributing author statement. If any of the co-authors have not filled in the statement, we will remind them to do so when the paper is revised. If all statements are not completed in a timely fashion this could hold up the re-review process. Should there be a problem getting one of your co-authors to fill in a statement we will be in contact. YOU MUST NOT ADD OR REMOVE AUTHORS UNLESS YOU HAVE ALERTED THE EDITOR HANDLING THE MANUSCRIPT TO THE CHANGE AND THEY SPECIFICALLY HAVE AGREED TO IT.**

**Response:** We confirm that we have contacted all co-authors and reminded them to fill in the contributing author statement.

23. **Please ensure that the paper adheres to the PLOS Data Availability Policy (see** [**https://can01.safelinks.protection.outlook.com/?url=http%3A%2F%2Fjournals.plos.org%2Fplosmedicine%2Fs%2Fdata-availability&data=05%7C02%7Cria.garg%40mail.utoronto.ca%7C0cfe141e8999421a910e08de3e5528d2%7C78aac2262f034b4d9037b46d56c55210%7C0%7C0%7C639016732647779801%7CUnknown%7CTWFpbGZsb3d8eyJFbXB0eU1hcGkiOnRydWUsIlYiOiIwLjAuMDAwMCIsIlAiOiJXaW4zMiIsIkFOIjoiTWFpbCIsIldUIjoyfQ%3D%3D%7C0%7C%7C%7C&sdata=9aXZCFPXWkHeEOm68BuXvwYGIR6kQZtbpqZUU3TegVo%3D&reserved=0)**](https://can01.safelinks.protection.outlook.com/?url=http%3A%2F%2Fjournals.plos.org%2Fplosmedicine%2Fs%2Fdata-availability&data=05%7C02%7Cria.garg%40mail.utoronto.ca%7C0cfe141e8999421a910e08de3e5528d2%7C78aac2262f034b4d9037b46d56c55210%7C0%7C0%7C639016732647779801%7CUnknown%7CTWFpbGZsb3d8eyJFbXB0eU1hcGkiOnRydWUsIlYiOiIwLjAuMDAwMCIsIlAiOiJXaW4zMiIsIkFOIjoiTWFpbCIsIldUIjoyfQ%3D%3D%7C0%7C%7C%7C&sdata=9aXZCFPXWkHeEOm68BuXvwYGIR6kQZtbpqZUU3TegVo%3D&reserved=0))**, which requires that all data underlying the study's findings be provided in a repository or as Supporting Information. For data residing with a third party, authors are required to provide instructions with contact information for obtaining the data. PLOS journals do not allow statements supported by "data not shown" or "unpublished results." For such statements, authors must provide supporting data or cite public sources that include it.**

**Response:** The dataset from this study is held securely in coded form at ICES. While legal data sharing agreements between ICES and data providers (e.g., healthcare organizations and government) prohibit ICES from making the dataset publicly available, access may be granted to those who meet pre-specified criteria for confidential access, available at www.ices.on.ca/DAS (email: das@ices.on.ca). The full dataset creation plan and underlying analytic code are also available from the ICES via email to [das@ices.on.ca](mailto:das@ices.on.ca) upon request to qualified applicants, understanding that the computer programs may rely upon coding templates or macros that are unique to ICES and are therefore either inaccessible or may require modification.

24. **Please review your reference list to ensure that it is complete and correct. If you have cited papers that have been retracted, please include the rationale for doing so in the manuscript text, or remove these references and replace them with relevant current references. Any changes to the reference list should be mentioned in the rebuttal letter that accompanies your revised manuscript.**

**Response:** We confirm that we have reviewed the reference list.
